# Anesthetic Propofol Promotes Tumor Metastasis in Lungs via GABA_A_R‐Dependent TRIM21 Modulation of Src Expression

**DOI:** 10.1002/advs.202102079

**Published:** 2021-07-15

**Authors:** Qidong Liu, Zhihao Sheng, Chun Cheng, Hui Zheng, Michael Lanuti, Rong Liu, Ping Wang, Yuan Shen, Zhongcong Xie

**Affiliations:** ^1^ Anesthesia and Brain Research Institute Shanghai Tenth People's Hospital School of Medicine Tongji University Shanghai 200072 P. R. China; ^2^ Department of Anesthesiology National Cancer Center/National Clinical Research Center for Cancer/Cancer Hospital Chinese Academy of Medical Sciences and Peking Union Medical College Beijing 100021 P. R. China; ^3^ Division of Thoracic Surgery Department of Surgery Massachusetts General Hospital and Harvard Medical School Boston MA 02114 USA; ^4^ Tongji University Cancer Center Shanghai Tenth People's Hospital School of Medicine Tongji University Shanghai 200072 P. R. China; ^5^ Geriatric Anesthesia Research Unit, Department of Anesthesia, Critical Care and Pain Medicine Massachusetts General Hospital and Harvard Medical School Charlestown MA 02129 USA

**Keywords:** GABA_A_R, lung metastasis, propofol, tumor

## Abstract

Generation of circulating tumor cells (CTCs), a key step in tumor metastasis, occurs during surgical tumor resection, often performed under general anesthesia. Propofol is the commonly used anesthetic, but its effects on CTCs and tumor metastasis remain largely unknown. Propofol effects are investigated in an experimental metastasis model by injecting tumor cells and, subsequently, low‐ or standard‐dose propofol to nude mice through tail vein. Propofol‐ or vehicle‐treated tumor cells are also injected to the mice. An in vitro tumor cell–vascular endothelial cell adhesion assay, immunofluorescence, and other methods are employed to assess how propofol affects tumor cell adhesion and extension. Propofol induces more lung tumor metastasis in mice than control. Mechanistically, propofol enhances tumor cell adhesion and extension through GABA_A_R to downregulate TRIM21 expression, leading to upregulation of Src, a protein associated with cell adhesion. These results demonstrate that propofol may promote tumor metastasis through GABA_A_R–TRIM21–Src mechanism.

## Graphical abstract

The commonly used anesthetic propofol activates GABA receptors to downregulate expression of TRIM21, a protein mediating tumor metastasis, and consequently to upregulate expression of Src, a protein regulating cell adhesion and extension, leading to the promotion of tumor metastasis in lungs of mice. These data suggest GABA receptor–TRIM21–Src–tumor cell adhesion/extension as the underlying mechanism of tumor metastasis.

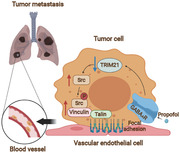

## 1 Introduction

Perioperative factors including anesthesia and surgery have been reported to associate with poor prognosis in tumor patients, including increased mortality and recurrence rates.^[^ ^1^ ^]^ Compared to regional anesthesia, general anesthesia is associated with a higher rate of tumor recurrence and mortality,^[^ ^2^ ^]^ but conflicting reports exist.^[^ ^3^ ^]^ A recent study assessing 196 303 patients with digestive tumor did not detect differences in tumor metastasis or recurrence in the patients who received different anesthetics.^[^ ^4^ ^]^

In particular, the effect of propofol, the commonly used anesthetic, on tumor prognosis is under debate. In some cases, propofol might improve tumor prognosis compared with other anesthetics, including sevoflurane.^[^ ^5^ ^]^ On the other hand, there was no significant difference in the count of generation of circulating tumor cells (CTCs) in patients undergoing surgical resection of breast cancer under propofol versus sevoflurane anesthesia.^[^ ^6^ ^]^ CTCs, which occurs during surgical tumor resection, are critical for tumor metastasis and recurrence.^[^ ^7^ ^]^ In addition, although some cell culture studies reported that propofol regulates proliferation, migration, and invasion of tumor cells,^[^ ^8^ ^]^ these in vitro studies employed a longer period (e.g., 24 to 72 h) or multiple administrations of propofol treatment. Therefore, it is important to establish more clinically relevant models to determine the effects of propofol on CTCs and tumor metastasis.

Recently, Li et al. systematically compared the difference of anesthetics on tumor metastasis and reported that inhalation anesthetic sevoflurane led to significantly more lung metastasis of breast cancer in mice after surgical resection of tumor as compared to propofol by changing the tumor microenvironment in lungs.^[^ ^9^ ^]^ However, the study did not demonstrate whether anesthetic itself (vs nonanesthesia condition) could promote or inhibit tumor progression. Therefore, it remains unknown whether standard‐dose propofol can promote tumor metastasis compared to low‐dose propofol or nonanesthesia condition.

To assess this, here, we employed an experimental metastasis mouse model, often used to study tumor metastasis,^[^ ^10^ ^]^ rather than a spontaneous metastasis model, to determine the effects of standard‐dose propofol on tumor metastasis as compared to low‐dose propofol or nonanesthesia condition. This is because although spontaneous metastasis models are valuable for in vivo tumor research, it is not possible to perform tumor resection with low‐dose propofol or nonanesthesia conditions in spontaneous metastasis models.

We injected tumor cells and, subsequently, low‐ or standard‐dose propofol into nude mice via the tail vein to conceptually mimic the condition by which propofol mixes with CTCs. We also injected propofol‐ or vehicle‐treated tumor cells in nude mice via the tail vein to further determine the effects of propofol versus nonanesthesia condition on tumor metastasis. These two methods could conceptually represent the clinical condition of general anesthesia with standard‐dose propofol versus regional anesthesia with or without low‐dose propofol for sedation.

Surgical tumor resection can generate CTCs in the blood.^[^ ^11^ ^]^ Tumor metastasis begins with local migration and invasion of cancer cells from their primary sites into blood (intravasation), and is followed by adhesion of CTCs to vascular endothelial cells (VECs); finally, CTCs leave the blood stream (extravasation).^[^ ^7^, ^12^ ^]^ During extravasation, CTCs adhere to VECs,^[^ ^10^, ^13^ ^]^ and extension of these tumor cells with membrane protrusions makes it easier for the cells to adhere to and further penetrate through VECs, exiting the blood vessel^[^ ^10^, ^14^ ^]^ to infiltrate and colonize different organs.^[^ ^15^ ^]^ Thus, adhesion and extension of CTCs to VECs are critical steps during tumor metastasis. In the present study, we specifically focused on investigating the effects of propofol on extravasation (e.g., adhesion and extension), but not intravasation (e.g., migration and invasion).

Propofol is a type‐A *γ*‐aminobutyric receptor (GABA_A_R) agonist.^[^ ^16^ ^]^ GABA_A_Rs exist in peripheral tissues^[^ ^17^ ^]^ and promote migration and invasion of some tumor cells.^[^ ^18^ ^]^ Therefore, we sought to determine whether propofol could promote tumor metastasis via enhancing adhesion and extension of tumor cells in vivo in nude mice and in vitro through action on GABA_A_R.

Other factors are important regulators of tumor metastasis. Src is a nonreceptor cytoplasmic kinase that mediates formation of focal adhesions^[^ ^19^ ^]^ and arrangement of actin filaments for cell extension,^[^ ^20^ ^]^ which can regulate tumor cell metastasis.^[^ ^21^ ^]^ Upregulation of Src expression critically contributes to Src activation,^[^ ^22^ ^]^ which can be detected in many tumors including colon, lung, breast, and endometrial tumors.^[^ ^23^ ^]^ Src can regulate migration and invasion of tumor cells.^[^ ^24^ ^]^ Activation of Src promotes breast tumor cell extravasation into the brain parenchyma.^[^ ^25^ ^]^ Inhibition of Src activity effectively reduces adhesion of colorectal cancer cell line SW620 and HT29 to fibroblasts.^[^ ^26^ ^]^ Notably, PP2, a specific inhibitor of Src activation, can reduce tumor metastasis.^[^ ^27^ ^]^ Further, Src can be quickly metabolized via ubiquitination.^[^ ^28^ ^]^ We, therefore, assessed whether propofol could act on GABA_A_R to regulate Src expression by affecting Src ubiquitination, consequently influencing tumor metastasis.

Additionally, members of the tripartite motif (TRIM) protein family contribute to tumor cell metastasis. TRIM21 suppresses progression of tumor metastasis by ubiquitylation and degradation of Snail^[^ ^29^ ^]^ and I*κ*B kinase *β*.^[^ ^30^ ^]^ In addition, TRIM21 can regulate adhesion of monocytes to endothelial cells.^[^ ^31^ ^]^ TRIM21 also interacts with cytoskeletal proteins. TRIM21 colocalizes with F‐actin, which can inhibit TRIM21 function.^[^ ^32^ ^]^ Therefore, it is possible that TRIM21 may regulate the effects of propofol on tumor metastasis, adhesion between tumor cells and endothelial cells, and cytoskeletal protein (e.g., F‐actin). However, whether propofol can act on GABA_A_R to regulate TRIM21 expression, leading to changes in Src expression and consequently alterations in adhesion and extension, remains unknown.

Therefore, the objective of the present study was to investigate the effects of propofol on tumor cell metastasis and the underlying mechanisms of these effects. We hypothesized that standard‐dose propofol promotes tumor metastasis as compared to low‐dose propofol or nonanesthesia condition through GABA_A_R–TRIM21–Src mechanism. We found that propofol activated GABA_A_R to decrease expression of TRIM21 and increase expression of Src, which enhanced tumor cell adhesion and extension, leading to promote tumor metastasis in lungs of mice.

## 2 Results

### 2.1 Propofol Promotes Tumor Cell Metastasis Mediated by GABA_A_R

**Figure 1:**
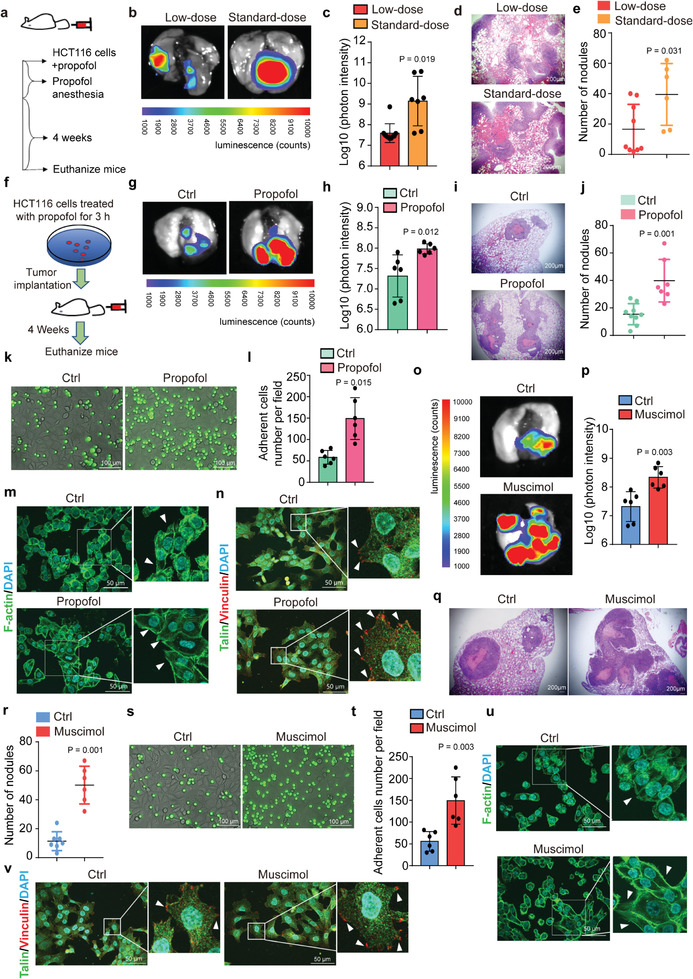
Propofol and GABA_A_R agonist muscimol promote tumor metastasis in the lungs of mice by enhancing tumor cell adhesion and extension. a) Study diagram: Injection of HCT116 cells overexpressing luciferase was followed by injection of low‐dose or standard‐dose propofol into the tail vein of BALB/c nude mice. b) Tumor metastasis in lungs of mice injected with low‐ versus standard‐doses of propofol, by ex vivo bioluminescent assay. c) Quantification of bioluminescent photon intensity (*N* = 10 in low‐dose group, *N* = 7 in standard‐dose group; mean ± SD, Student's *t*‐test, *P* = 0.019). d) H&E staining of metastatic lung nodules (scale bar = 200 µm). e) Quantification of metastatic nodules (*N* = 9 in low‐dose group, *N* = 6 in standard‐dose group; median and IQR, Mann–Whitney test, *P* = 0.031). f) Study diagram: HCT116 cells overexpressing luciferase were treated with propofol for 3 h (mimicking average clinical anesthesia time) and were injected intravenously into BALB/c nude mice. g) Ex vivo bioluminescent assay comparing lung metastasis of mice injected with propofol‐treated or DMSO‐treated (nonanesthesia control condition) cells. h) Quantification of bioluminescent photon intensity (*N* = 6 in each group; mean ± SD, Student's *t*‐test, *P* = 0.012). i) H&E staining of metastatic lung nodules (scale bar = 200 µm). j) Quantification of metastatic nodules (*N* = 9 in ctrl group, *N* = 7 in propofol group; median and IQR, Mann–Whitney test, *P* = 0.001). k) Fluorescent micrographs showing adhesion of propofol‐treated or DMSO‐treated HCT116 cells (green) to the HUVEC monolayer (scale bar = 100 µm). l) Quantification of the HCT116 cells adhesion to the HUVEC monolayer under static conditions (*N* = 6, mean ± SD, Student's *t*‐test, *P* = 0.015). m) F‐actin labeling of cell membrane protrusions in propofol‐treated or control HCT116 cells (F‐actin: phalloidin; nucleus: DAPI; scale bar = 50 µm). Arrows indicate cell membrane protrusion extensions. n) Immunofluorescence staining of focal adhesion proteins Talin and Vinculin in propofol‐treated HCT116 cells compared to control cells (scale bar = 50 µm). Arrows indicate focal adhesions. o) Ex vivo bioluminescent assay of lung metastasis of mice injected with muscimol‐treated cells compared to control cells. p) Quantification of bioluminescent photon intensity (*N* = 6 in each group; mean ± SD, Student's *t*‐test, *P* = 0.003). q) H&E staining of metastatic nodules (scale bar = 200 µm). r) Quantification of metastatic nodules (*N* = 7 in ctrl group, *N* = 6 in muscimol group; median and IQR, Mann–Whitney test, *P* = 0.001). s) Fluorescent micrographs showing adherence of HCT116 cells (green) treated with muscimol or control conditions to the HUVEC monolayer (scale bar = 100 µm). t) Quantification of the HCT116 cells adhesion to an HUVECs monolayer (*N* = 6, mean ± SD, Student's *t*‐test, *P* = 0.003). u) F‐actin labeling of cell extensions in HCT116 cells treated with muscimol compared to controls (F‐actin: phalloidin; nucleus: DAPI; scale bar = 50 µm). Arrows indicate cell membrane protrusion extensions. v) Immunofluorescence staining of Talin and Vinculin in HCT116 cells treated with muscimol or controls (scale bar = 50 µm). Arrows indicate focal adhesions. Ctrl, control; HUVEC, human umbilical vein endothelial cell; H&E, hematoxylin and eosin; SD, standard deviation.

We injected HCT116 cells and, subsequently, a low or standard dose of propofol into nude mice via the tail vein (**Figure** **1**a). Ex vivo bioluminescence imaging of mouse lungs four weeks after injections showed that treatment with standard‐dose propofol (240 mg kg^−1^ h^−1^ over 1 h) led to significant increases in tumor metastasis, represented by photon intensity (7.59 ± 0.43 vs 9.14 ± 1.11, *P* = 0.019, Student's *t*‐test) (Figure 1b,c) and number of nodules (16.55 ± 15.49 vs 39.6 ± 18.57, *P* = 0.031, Student's *t*‐test) (Figure 1d,e) as compared to treatment with low‐dose propofol (20 mg kg^−1^). These data suggest that propofol may promote lung metastasis of tumor cells in vivo.

Next, we compared the effects of propofol anesthesia versus the nonanesthesia condition on tumor metastasis by injecting propofol‐treated versus vehicle (DMSO)‐treated HCT116 cells into nude mice via the tail vein (Figure 1f). We found that the propofol‐treated tumor cells had increased photon intensity (7.31 ± 0.47 vs 7.98 ± 0.16, *P* = 0.019, Student's *t*‐test) (Figure 1g,h) and number of nodules (15.44 ± 7.21 vs 42.33 ± 14.62, *P* = 0.001, Student's *t*‐test) (Figure 1i,j) as compared to DMSO‐treated tumor cells (vehicle control condition) four weeks after injection. These data further suggest that propofol may promote tumor metastasis to lungs in the mice.

In vitro, treatment with propofol caused more HCT116 cells to adhere to a monolayer of human umbilical vein endothelial cells (HUVECs) than control (Figure 1k,l). Propofol also induced greater cell membrane protrusion extensions with more apparent F‐actin structures (Figure 1m). Propofol increased focal adhesion sites detected by immunofluorescence staining of focal adhesion proteins Talin and Vinculin (Figure 1n; Figure S1a, Supporting Information), further demonstrating that propofol increased adhesion of HCT116 cells. Propofol also enhanced adhesion and extension of other tumor cell lines, including mouse colorectal tumor cell line CT26 (Figure S1b–d, Supporting Information), breast tumor cell line MDA‐MB‐231 (Figure S1e–g, Supporting Information), human nonsmall‐cell lung carcinoma cell line A549 (Figure S1h–j, Supporting Information), and endometrial carcinoma cell line Ishikawa (Figure S1k–m, Supporting Information).

Propofol is a GABA_A_R agonist. We found that GABA_A_R antagonist bicuculine induced depolarization of membrane potential in HCT116 cells, indicating the cells possess functional GABA_A_R (Figure S2a,b, Supporting Information). Further, like propofol, the GABA_A_R agonist muscimol‐treated HCT116 cells also increased lung metastasis of tumor compared to control HCT116 cells (Figure 1o–r) in nude mice. Consistently, in vitro studies revealed that muscimol enhanced adhesion and extension of HCT116 cells (Figure 1s–v; Figure S3a, Supporting Information) as well as other tumor cell lines, such as CT26 (Figure S3b, Supporting Information), MDA‐MB‐231 (Figure S3c, Supporting Information), A549 (Figure S3d, Supporting Information), and Ishkawa (Figure S3e, Supporting Information), as compared to control condition.

Treatment of HCT116 cells with GABA_A_R antagonist bicuculine or benzodiazepine antagonist flumazenil in vitro decreased cell adhesion, and benzodiazepine agonist diazepam increased cell adhesion and extension (Figure S4a–e, Supporting Information). We further confirmed that GABA_A_R antagonist bicuculine attenuated the propofol‐induced enhancement of HCT116 cell adhesion and extension (Figure S5a–e, Supporting Information). Further, knockout (KO) of both GABA_A_R subunit *β*3 and *δ* (Figure S5f, Supporting Information) decreased the general adhesion and extension ability of HCT116 and specifically attenuated the propofol‐promoted adhesion and extension of HCT116 (Figure S5g–i, Supporting Information). However, NMDA receptor antagonist MK‐801 and acetylcholine receptor antagonist benzethonium did not regulate adhesion of HCT116 cells (Figure S6a,b, Supporting Information). These data suggest that propofol promotes metastasis of tumor cells by enhancing their adhesion and extension, specifically via acting on GABA_A_R.

### 2.2 Propofol Enhances Cell Adhesion and Extension by Regulating GABA_A_R‐Dependent Src Ubiquitination

**Figure 2:**
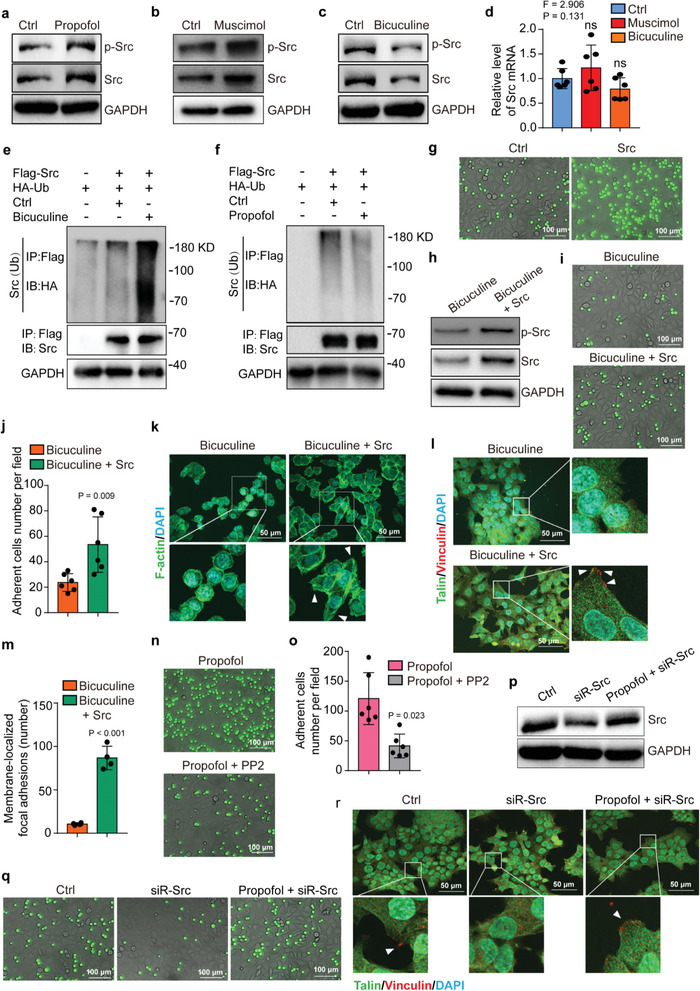
Propofol enhances adhesion and extension of HCT116 cells through GABA_A_R‐dependent Src expression. Western blot analysis of Src and p‐Src expression in HCT116 cells treated with a) propofol, b) muscimol, or c) bicuculine. GAPDH is a loading control. d) qRT‐PCR results for *Src* mRNA levels in HCT116 cells treated with muscimol, bicuculine, or control conditions (*N* = 6 in each group, mean ± SD, one‐way ANOVA test with post‐hoc Bonferroni test, *F* = 2.906, *P* = 0.131). Western blot analysis of Src ubiquitylation in HCT116 cells overexpressing Src‐Flag or control vector and treated with e) bicuculine or f) propofol compared to control condition. Src was immunoprecipitated with anti‐Flag and immunoblotted with anti‐HA. g) Fluorescent micrographs showing adhesion between the HUVEC monolayer and the HCT116 cells (green) with or without Src overexpression (scale bar = 100 µm). h) Western blot analysis of Src and p‐Src expression in bicuculine‐treated HCT116 cells with or without Src overexpression. GAPDH is a loading control. i) Fluorescent micrographs showing adhesion between HUVEC monolayer and the bicuculine‐treated HCT116 cells (green) with or without Src overexpression (scale bar = 100 µm). j) Quantification of the HCT116 cell adhesion to the HUVEC monolayer (*N* = 6, mean ± SD, Student's *t*‐test, *P* = 0.009). k) F‐actin labeling of membrane protrusions in bicuculine‐treated HCT116 cells with or without Src overexpression (scale bar = 100 µm). Arrows indicate extensions of cell membrane protrusions. l) Immunofluorescence staining of Talin and Vinculin in the bicuculine‐treated HCT116 cells with or without Src overexpression (scale bar = 50 µm). Arrows indicate focal adhesions. m) Quantification of the membrane‐localized Talin and Vinculin in focal adhesions (*N* = 4, mean ± SD, Student's *t*‐test, *P* < 0.001). n) Fluorescent micrographs showing adhesion between the HUVEC monolayer and the propofol‐treated HCT116 cells (green) with or without Src inhibitor PP2 treatment (scale bar = 100 µm). o) Quantification of panel (n) (*N* = 6, mean ± SD, Student's *t*‐test, *P* = 0.023). p) Western blot analysis of Src expression following RNAi knockdown of Src in HCT116 cells treated with propofol or control condition. GAPDH is a loading control. q) Fluorescent micrographs showing adhesion between the HUVEC monolayer and the HCT116 cells with knockdown of Src (green) treated with propofol or control condition (scale bar = 100 µm). r) Immunofluorescence staining of Talin and Vinculin in HCT116 cells with knockdown of Src treated with propofol or control condition (scale bar = 50 µm). Arrows indicate focal adhesions. Ctrl, control; HUVEC, human umbilical vein endothelial cell; SD, standard deviation; p‐Src, phosphorylated Src.

Propofol (**Figure** **2**a) and muscimol (Figure 2b) treatments upregulated the amounts of Src and phosphorylated Src (p‐Src) expressed in HCT116 cells, whereas bicuculine treatment (Figure 2c) downregulated expression of Src and p‐Src. However, neither muscimol nor bicuculine significantly changed the expression of *Src* mRNA (Figure 2d). Further, bicuculine (Figure 2e) promoted but propofol (Figure 2f) and muscimol (Figure S7a, Supporting Information) inhibited ubiquitylation of Src. Knockout of GABA_A_R subunit *β*3 and *δ* decreased the Src protein level and blocked the function of propofol on upregulating the expression of Src and p‐Src (Figure S7b,c, Supporting Information). Furthermore, the experimental half‐life of Src protein was markedly prolonged (estimated from 4 to 8 h in the present study) with the treatment of muscimol or propofol in the cycloheximide (CHX) chase assay even at 12 h after the propofol treatment (Figure S7d, Supporting Information). Treatment of HCT116 cells with PYR‐41, an inhibitor of ubiquitin‐activating enzyme (E1), upregulated Src and p‐Src expression (Figure S8a, Supporting Information) and promoted cell adhesion and extension (Figure S8b–f, Supporting Information). Consistently, overexpression of Src (Figure S9a, Supporting Information) promoted HCT116 cell adhesion and extension (Figure 2g; Figure S9b–e, Supporting Information). Treatment of HCT116 cells with Src inhibitor PP2 (10 × 10^−6^ m) reduced Src and p‐Src expression (Figure S10a, Supporting Information) and decreased cell adhesion and extension (Figure S10b–f, Supporting Information). Similarly, PP2 decreased the adhesion of human colorectal cancer cell (HT‐29) (Figure S10g, Supporting Information) and mouse colorectal cancer cell (CT26) on HUVECs (Figure S10h, Supporting Information). Mechanistically, PP2 attenuated the PYR‐41‐induced upregulation of Src and p‐Src (Figure S10i, Supporting Information) and the PYR41‐induced enhancement of HCT116 cell adhesion and extension (Figure S10j–m, Supporting Information). Focal adhesion kinase (FAK) forms a complex with Src in tumor cells to regulate growth and metastasis. However, propofol did not significantly regulate phosphorylation of FAK (Figure S10n, Supporting Information). These data suggest FAK may not contribute to the propofol‐promoted tumor metastasis.

Further, overexpression of Src restored the bicuculine‐induced reduction of Src and p‐Src (Figure 2h) and attenuated the bicuculine‐induced inhibition of HCT116 cell adhesion and extension (Figure 2i–m). Moreover, PP2 attenuated the propofol‐induced enhancement of adhesion and extension in HCT116 cells (Figure 2n,o, Figure S11a–c, Supporting Information) as well as in other cells, including HT‐29 and CT26 cells (Figure S11d,e, Supporting Information). RNAi knockdown of Src (Figure 2p) repressed the adhesion ability of HCT116 cells and blocked the propofol‐promoted adhesion and extension (Figure 2q,r; Figure S11f,g, Supporting Information). These data suggest that propofol inhibits Src ubiquitination by acting on GABA_A_R, leading to accumulation of Src, which then promotes tumor cell adhesion and extension.

### 2.3 GABA_A_R Upregulates Src via TRIM 21

**Figure 3:**
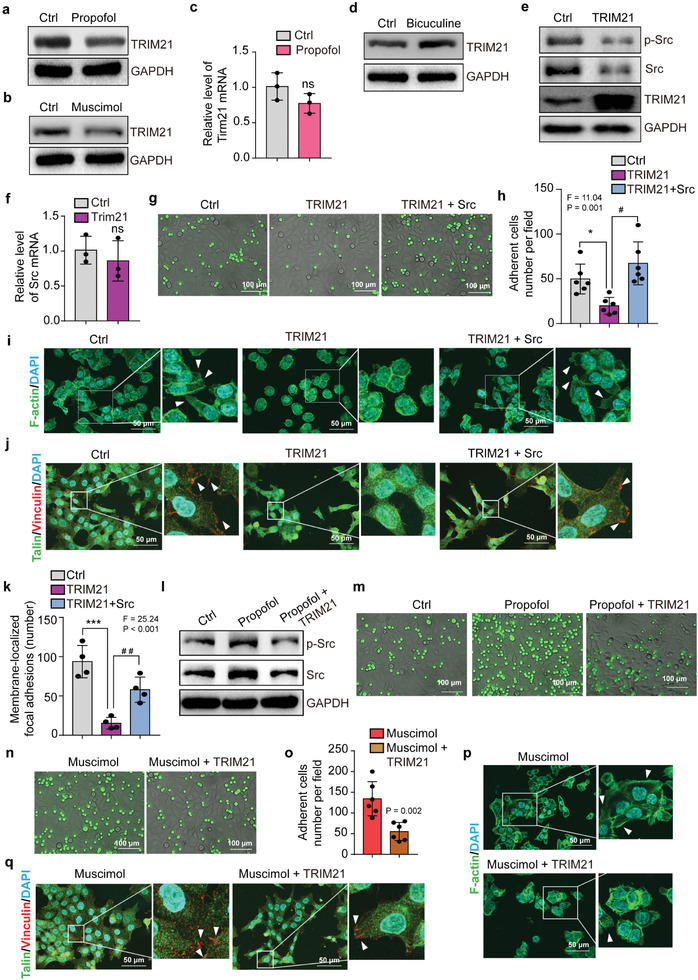
TRIM21 regulates Src protein level to mediate GABA_A_R signaling. Western blot analysis of TRIM21 expression in HCT116 cells treated with a) propofol, b) muscimol, or d) bicuculine. c) qRT‐PCR analysis of *TRIM21* mRNA expression in HCT116 cells treated with propofol or control condition (*N* = 3, mean ± SD, Student's *t*‐test, *P* > 0.5). e) Western blot analysis of Src and p‐Src expression in HCT116 cells with or without overexpression of TRIM21. GAPDH is a loading control. f) qRT‐PCR analysis of *Src* mRNA expression in HCT116 cells with or without overexpression of TRIM21 (*N* = 3, mean ± SD, Student's *t*‐test, *P* > 0.5). g) Fluorescent micrographs of adhesion between the HUVEC monolayer and the HCT116 cells (green) with or without overexpression of TRIM21 and Src (scale bar = 100 µm). h) Quantification of the HCT116 cells adhesion to the HUVECs monolayer (*N* = 6, mean ± SD, one‐way ANOVA test with post‐hoc Bonferroni test, *F* = 11.04, *P* = 0.001). i) F‐actin labeling of membrane protrusions in HCT116 cells with or without overexpression of TRIM21 and Src (scale bar = 100 µm). Arrows indicate extensions of cell membrane protrusions. j) Immunofluorescence staining of Talin and Vinculin in HCT116 cells with or without overexpression of TRIM21 and Src (scale bar = 50 µm). Arrows indicate focal adhesions. k) Quantification of the membrane‐localized Talin and Vinculin in focal adhesion (*N* = 4, mean ± SD, one‐way ANOVA test with post‐hoc Bonferroni test, *F* = 25.24, *P* < 0.001). l) Western blot analysis of Src and phosphorylated Src (p‐Src) expression in HCT116 cells with or without overexpression of TRIM21 and following propofol or control condition. GAPDH is a loading control. m) Fluorescent micrographs of the adhesion between the HUVEC monolayer and the HCT116 cells (green) with or without overexpression of TRIM21 and treated with propofol or control condition (scale bar = 100 µm). n) Fluorescent micrographs showing adhesion of muscimol‐treated HCT116 cells (green) with or without TRIM21 overexpression to the HUVEC monolayer (scale bar = 100 µm). o) Quantification of panel (n) (*N* = 6, mean ± SD, Student's *t*‐test, *P* = 0.002). p) F‐actin labeling of membrane protrusions in muscimol‐treated HCT116 cells with or without TRIM21 overexpression (scale bar = 100 µm). Arrows indicate extensions of cell membrane protrusions. q) Immunofluorescence staining of Talin and Vinculin in muscimol‐treated HCT116 cells with or without TRIM21 overexpression (scale bar = 50 µm). Arrows indicate focal adhesions. Ctrl, control. *P < 0.05, ***P < 0.001, ^#^ *P* < 0.05, and ^##^ *P* < 0.01. Ctrl, control; HUVEC, human umbilical vein endothelial cell; SD, standard deviation; p‐Src, phosphorylated Src.

Treatment of HCT116 cells with propofol (**Figure** **3**a; Figure S12a, Supporting Information) and muscimol (Figure 3b; Figure S12b, Supporting Information) decreased TRIM21 protein amounts. But propofol did not regulate transcription of TRIM21 (Figure 3c). Bicuculine upregulated expression of TRIM21 (Figure 3d; Figure S12c, Supporting Information). Overexpression of TRIM21 downregulated Src (Figure 3e; Figure S12d, Supporting Information), but did not significantly change expression of *Src* mRNA (Figure 3f). Overexpression of TRIM21 also promoted ubiquitylation of Src (Figure S12e, Supporting Information). Finally, Co‐immunoprecipitation (Co‐IP) assay did not demonstrate the direct interaction between E3 ligase‐TRIM21 and Src (Figure S12f, Supporting Information). Conversely, RNAi knockdown of TRIM21 in HCT116 cells upregulated Src and p‐Src expression (Figure S13a, Supporting Information) and promoted cell adhesion and extension (Figure S13b–f, Supporting Information). Importantly, overexpression of TRIM21 attenuated HCT116 cell adhesion and extension, which was restored by overexpression of Src (Figure 3g–k).

Overexpression of TRIM21 also attenuated the propofol‐induced upregulation of Src and promotion of adhesion of HCT116 cells on HUVECs (Figure 3l,m). Overexpression of TRIM21 attenuated the muscimol‐induced enhancement of HCT116 cell adhesion and extension (Figure 3n–q; Figure S14, Supporting Information). These data suggest GABA_A_R activation increases Src by reducing TRIM21, leading to enhancement of adhesion and extension in HCT116 cells.

### 2.4 Inhibition of Src Attenuates Propofol‐ or Muscimol‐Promoted Tumor Metastasis in the Lungs

**Figure 4:**
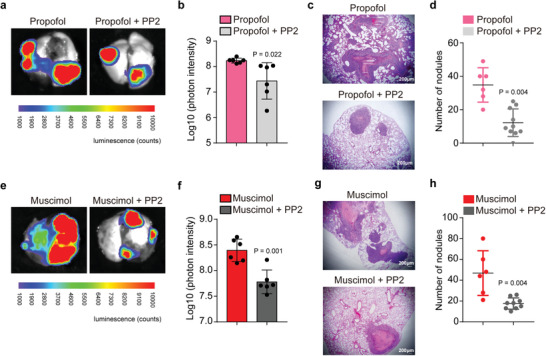
Inhibition of Src attenuates the propofol‐ or GABA_A_R agonist‐promoted tumor metastasis in mice lungs. a) Ex vivo bioluminescent assay of lung tumor metastasis. HCT116 cells overexpressing luciferase were treated with propofol or propofol plus PP2 (inhibitor of Src) for 3 h. Treated cells were then injected intravenously into BALB/c nude mice through the tail vein. b) Quantification of bioluminescent photon intensity (*N* = 6 in each group; mean ± SD, Student's *t*‐test, *P* = 0.022). c) H&E staining of metastatic lung nodules (scale bar = 200 µm). d) Quantification of metastatic nodules (*N* = 6 in the propofol group, *N* = 10 in the propofol plus PP2 group; median and IQR, Mann–Whitney test, *P* = 0.004). e) Ex vivo bioluminescent assay of lung tumor metastasis. HCT116 cells overexpressing luciferase were treated with muscimol or muscimol plus PP2 for 3 h. Treated cells were then injected intravenously into BALB/c nude mice. f) Quantification of bioluminescent photon intensity (*N* = 6 in each group; mean ± SD, Student's *t*‐test, *P* = 0.001). g) H&E staining of metastatic lung nodules (scale bar = 200 µm). h) Quantification of metastatic nodules (*N* = 6 in the muscimol group, *N* = 9 in the muscimol plus PP2 group; median and IQR, Mann–Whitney test, *P* = 0.004). VEC, vein endothelial cell; H&E, hematoxylin and eosin; SD, standard deviation.

Finally, we used a pharmacological but not a genetic approach to determine the more clinically relevant effects of inhibition of Src on propofol's effects. Ex vivo bioluminescent assays showed that treatment with PP2, an inhibitor of Src, significantly attenuated the propofol‐induced increases in the number of tumor nodules in mouse lungs (**Figure** **4**a,b). Histological analysis further confirmed that PP2 significantly attenuated the propofol‐promoted metastasis of HCT116 cells in mice lungs (Figure 4c,d). Ex vivo bioluminescent and histological analysis also confirmed that PP2 significantly attenuated the muscimol‐promoted metastasis of HCT116 cells in mice (Figure 4e–h). These data suggest that PP2 can inhibit the effects of propofol on tumor cell metastasis in mouse lungs.

**Figure 5:**
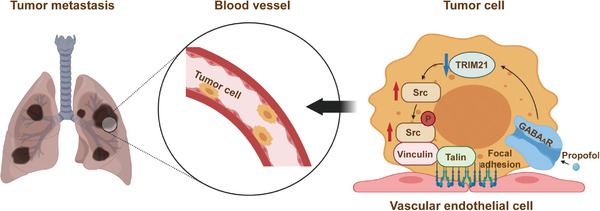
The hypothesized pathway indicating propofol promotes tumor metastasis. The hypothesized pathway indicating that propofol activated GABA_A_R to downregulate TRIM21 and consequently upregulate Src, leading to enhancement of adhesion and extension of the tumor cells with VECs and promotion of tumor metastasis in lungs of mice. Schematic created at BioRender.com with permission.

Collectively, these results suggest a potential pathway wherein propofol activated GABA_A_R, which caused downregulation of TRIM21 and consequent upregulation of Src, leading to enhancement of adhesion and extension of the tumor cells with VECs, potentially promoting tumor metastasis (**Figure** **5**).

## 3 Discussion

The data in this proof‐of‐concept study demonstrate that intravenous administration of standard‐dose propofol was associated with greater tumor metastasis in the lungs of nude mice as compared to low‐dose propofol or nonanesthesia condition. Mechanistically, propofol enhanced adhesion and extension of tumor cells to VECs, a critical step in tumor metastasis,^[^ ^33^ ^]^ by acting on GABA_A_R. Activation of GABA_A_R decreased expression of the cytosolic ubiquitin ligase TRIM21, leading to increases in the expression of Src, a protein associated with cell adhesion with VECs.

We showed that treatment with standard‐dose propofol promoted tumor metastasis to the lungs as compared to low‐dose propofol in nude mice (Figure 1a–e). We compared the effects of low versus standard dose of propofol on tumor metastasis by injecting different amounts of propofol via tail vein. However, this setting could not allow us to determine the effects of propofol anesthesia versus nonanesthesia condition because we could not use the pump to administer the same volume (600 µL) of intralipid (the vehicle of propofol) via tail vein without general anesthesia over 1 h. The injection of HCT116 cells without intralipid to mice would cause confounding influence because no vehicle of propofol was administered to the mice. We, therefore, used low‐dose propofol as the control condition in the experiment, which also represented the clinical condition for patients receiving standard‐dose propofol for general anesthesia or low‐dose propofol for sedation during regional anesthesia.

To compare the effects of propofol anesthesia versus nonanesthesia condition on tumor metastasis, we established another system by pretreating the tumor cells with propofol dissolved in DMSO versus DMSO (vehicle) alone for 3 h. We then injected these pretreated tumor cells into mice via the tail vein. We found that propofol‐treated tumor cells also resulted in greater metastasis in lungs of the nude mice as compared to the vehicle‐treated tumor cells (Figure 1f–j). These results further suggest that propofol would promote tumor metastasis in mice as compared to nonanesthesia condition.

Mechanistically, propofol enhanced adhesion and extension of tumor cells to VECs, a critical step in tumor metastasis,^[^ ^33^ ^]^ by acting on GABA_A_R. Activation of GABA_A_R decreased expression of the cytosolic ubiquitin ligase TRIM21, increasing expression of Src, a protein associated with cell adhesion with VECs.

Given that our objective was to determine the effects of standard‐dose propofol on tumor metastasis as compared to low‐dose propofol or nonanesthesia condition, the experimental metastasis model was the most suitable choice. While spontaneous metastasis models are valuable for in vivo research, in such mouse models, it is not possible to perform tumor resection with low‐dose propofol or nonanesthesia conditions. The experimental metastasis mouse model circumvents this limitation by injecting tumor cells and propofol to the mice via tail vein.

Propofol is a commonly used anesthetic in patients undergoing surgery, and surgical tumor resection may be associated with generation of CTCs,^[^ ^11^ ^]^ a critical step for tumor metastasis. However, previous studies focused on assessing the effects of propofol on tumor cell proliferation, migration, and invasion^[^ ^8^, ^34^ ^]^ but not on CTCs adhesion. In the present study, we specifically assessed the effects of propofol on CTCs by mixing the tumor cells with propofol in the blood of mice through injecting the tumor cells immediately before injecting propofol into the mouse tail vein (Figure 1). We also mixed tumor cells with propofol in cell culture medium before injecting the propofol‐treated cells to the mice via tail vein (Figure 1).

A recent study illustrated that propofol led to less lung metastasis than inhalational anesthetic sevoflurane in both syngeneic murine 4T1 and xenograft human MDA‐MB‐231 breast cancer models, demonstrating the effects of different anesthetics on tumor metastasis.^[^ ^9^ ^]^ Specifically, sevoflurane promoted the lung metastasis by modulating inflammatory cytokines and activating STAT3 pathway in the lungs.^[^ ^9^ ^]^ However, the study did not compare the difference of standard‐dose anesthetic (e.g., propofol) versus a low dose of the same anesthetic or nonanesthesia (control condition) on tumor metastasis. Moreover, this study determined the effects of anesthetics on the microenvironment of tumor, but not tumor cells themselves, as the underlying mechanisms.

In contrast, the present study, by employing both pharmacological and genetic regulation of the expression of GABA_A_R and TRIM21, revealed that propofol acted on GABA_A_R‐dependent TRIM21 modulation of Src expression of the tumor cells to lead to more lung metastasis as compared to nonanesthesia control condition. These findings will promote future in vivo approaches (e.g., employment of two‐photon microscopy to visualize the acute dynamic process of cell adhesion) to further reveal the role of GABA_A_R, TRIM21, and Src, in the effects of propofol on tumor metastasis in vivo.

Another study reported that propofol (2–10 µg mL^−1^ for 6 h) promotes migration and invasion of oral squamous carcinoma cells;^[^ ^34a^ ^]^ Meng et al. also reported that propofol (2–10 µg mL^−1^ for 1–12 h) increases proliferation and migration of human breast tumor cells;^[^ ^8c^ ^]^ and Garib et al. showed that propofol (6 µg mL^−1^) enhances migration of breast carcinoma cells.^[^ ^34b^ ^]^ Consistently, our results showed that propofol‐treated cells adhered to VECs more easily, had more focal adhesion sites, and demonstrated greater extension, promoting tumor progression.

Activation of Src contributes to epithelial–mesenchymal transition (EMT), which occurs during intravasation.^[^ ^35^ ^]^ Src can phosphorylate focal adhesion proteins^[^ ^36^ ^]^ to further regulate tumor metastasis.^[^ ^37^ ^]^ In addition, Src can promote mesenchymal–epithelial transition (MET)‐related processes. Inhibition of Src activity suppresses adhesion capacity of lung cancer A549 cells^[^ ^38^ ^]^ and inhibits adhesion of colorectal cancer cell line SW620 and HT29 to fibroblasts.^[^ ^26^ ^]^ Thus, Src can regulate both EMT‐ and MET‐related processes in tumor cells. Accordingly, we found that decreasing Src inhibited the propofol‐promoted tumor cell adhesion and extension.

Interestingly, PP2, a Src kinase inhibitor, decreased both Src and phosphorylated Src amounts in the present study. The underlying mechanism of such observation is not known at present. We postulated that reduction in phosphorylated Src could lead to increased degradation of Src as some sites of protein phosphorylation could block the action of degradation enzyme of the protein.^[^ ^39^ ^]^ Consistently, a previous study also demonstrated that PP2 decreased both Src and phosphorylated Src protein levels.^[^ ^40^ ^]^ Moreover, we did not demonstrate the direct interaction between E3 ligase‐TRIM21 and Src in the Co‐IP assay, suggesting that the degradation of Src could be indirectly regulated by TRIM21.

Adhesion of tumor cells occurs within a short period of time,^[^ ^14b^ ^]^ suggesting that the regulatory process should be quick. Ubiquitination is a rapid process regulating protein expression.^[^ ^41^ ^]^ In line with this, we found that propofol or activation of GABA_A_R increased Src expression by inhibiting Src ubiquitination to promote adhesion and extension of tumor cells to VECs. Moreover, colocalization of GABA_A_R and focal adhesion protein confirmed a direct path of close spatial regulation. Finally, Src inhibitor PP2 blocked GABA_A_R or propofol‐mediated lung metastasis (Figure 4). These results reveal that propofol might enhance adhesion and extension of tumor cells via GABA_A_R‐regulated changes in Src expression. We also showed that TRIM21 could be regulated by propofol and GABA_A_R agonist. TRIM21 regulated Src expression and, consequently, tumor cell adhesion and extension. Taken together, these data suggest a potential propofol–GABA_A_R–TRIM21–Src–tumor cell adhesion/extension mechanism. Future studies are warranted to further test whether the presence of such a cascade is responsible for propofol‐promoted tumor metastasis.

On the other hand, several studies demonstrated that propofol inhibits tumor cell progression.^[^ ^42^ ^]^ Zhang et al. reported that continuous exposure to propofol (8 µg mL^−1^, every week) inhibits growth of xenografts of primary colorectal tumors.^[^ ^34c^ ^]^ Wang et al. showed that invasion, migration, and growth of malignant pheochromocytoma cells can be inhibited by propofol (1–10 µg mL^−1^) treatment for 24–48 h. They also found that propofol (35 mg kg^−1^) could inhibit growth of malignant pheochromocytoma tumors in a xenograft model.^[^ ^34d^ ^]^ The reason for these contrasting findings is not yet known. However, these studies differed from present study in several ways: 1) they did not assess the effects of propofol on tumor metastasis and only used a subcutaneously injected xenograft model; 2) multiple administrations of propofol were used in some studies; 3) treatment times were generally longer.

Several studies demonstrated that propofol can attenuate the proliferation, invasion, and migration of tumor cells.^[^ ^43^ ^]^ However, most of these studies were performed in vitro with long duration of propofol treatment, including 24,^[^ ^43c,e^ ^]^, 48,^[^ ^43a,d^ ^]^ and 72 h.^[^ ^43b^ ^]^ In the present study, we only treated the tumor cells with propofol for 3 h, mimicking common surgery time in patients, for both in vitro (adhesion and extension of tumor cells) and in vivo (tumor metastasis) experiments. Moreover, injection of tumor cells followed by injection of standard‐dose propofol also increased tumor metastasis as compared to injection of tumor cells followed by injection of low‐dose propofol in mice.

The present study has several strengths. First, we established a system to conceptually mimic the clinical situation in which propofol encounters CTCs. Second, we showed that intravenous administration of propofol promoted tumor metastasis to lungs in mice by using experimental metastasis model. Finally, we revealed a GABA_A_R–TRIM21–Src–tumor cell adhesion/extension may be the underlying mechanism of this propofol‐promoted tumor metastasis.

The present study also has several limitations. First, we did not inject the GABA_A_R knockout or TRIM21 overexpression HCT116 cells into the mice to further determine the role of GABA_A_R or TRIM21 in the propofol‐promoted tumor metastasis in vivo. This is because TRIM21 overexpression or GABA_A_R knockout itself could have long lasting effects in regulating tumor metastasis even after the clearance of propofol from blood, thus generating potential confounding influence. However, the in vitro data demonstrated the role of GABA_A_R or TRIM21, by using genetic modifications, in the propofol‐promoted tumor cell adhesion to VECs. Future studies using genetically modified tumor cells and two‐photon microscopy to visualize the acute dynamic process of cell adhesion will allow us to further determine whether propofol can promote adhesion of CTCs to VECs via interactions of GABA_A_R, TRIM21, and Src in vivo, leading to penetration of the vascular endothelium to start the process of extravasation. Second, we did not compare the effects of propofol with other anesthetics on tumor metastasis, as demonstrated in another study.^[^ ^9^ ^]^ However, the objective of the present study was to illustrate that standard‐dose anesthetic could promote tumor metastasis in mice as compared to low‐dose anesthetic or nonanesthesia control condition. Third, the administration of low‐dose propofol (20 mg kg^−1^) was once, but the administration of standard‐dose propofol (240 mg kg^−1^) was over 1 h. This was because the mouse would move around under the low‐dose propofol and unable to receive 1 h infusion of propofol. Thus, we did a single injection of low‐dose propofol to the mouse.

In conclusion, this study demonstrated that the commonly used anesthetic propofol promoted tumor metastasis in mouse lungs. Mechanistically, propofol could promote tumor metastasis by enhancing tumor cell adhesion and extension via interactions of GABA_A_R, TRIM21, and Src. Finally, inhibition of Src might mitigate the propofol‐promoted tumor metastasis. These findings will likely foster more research on anesthesia and tumor metastasis in preclinical and clinical settings, ultimately leading to better patient prognosis after surgical resection of tumors.

## 4 Experimental Section

### Mice

6‐week‐old female BALB/c nude mice (Charles River, China) were employed. The animal protocol was approved by the Standing Committee on Animals at Shanghai Tenth People's Hospital and mice were maintained in accordance with federal guidelines in the Laboratory Animal Center at Tongji University (protocol number: SHDSYY‐2018‐17310101). All mice were raised in sterilized cages under pathogen‐free conditions (22–26 °C, 12/12 light/dark cycle) and offered food and water ad libitum. Animal suffering during euthanasia was ameliorated by using CO_2_ overdose followed by cervical dislocation. Efforts were made to minimize the number of animals used in the studies. The manuscript was written according to ARRIVE guidelines.

### Generation of HCT116 Cells Overexpressing Luciferase

Generation of tumor cells with stable overexpression of luciferase enables ex vivo bioluminescence imaging of tumors. Thus, the HCT116 cells were generated with stable overexpression of luciferase to visualize tumors in mouse lung. HCT116 cells were infected with pSLenti‐EF1‐Luc2‐F2A‐Puro virus (Oobio, Shanghai, China). Stable cell lines were selected by adding 1 µg mL^−1^ puromycin (S7417, Selleck, Houston, TX, USA) to culture medium. The cells were digested with 0.25% trypsin (Gibco, Grand Island, NY, USA). A total of 1.5 million cells were centrifugally collected and resuspended in 100 µL culture medium for the injection to mice.

### Administration of Low‐Dose or Standard‐Dose Propofol in Mice

The effects of propofol on tumor metastasis were determined by injecting a low dose (20 mg kg^−1^ once, about 0.5 mg propofol in 50 µL intralipid per mouse) versus standard dose (240 mg kg^−1^ h^−1^ for 1 h, about 6 mg propofol in 600 µL intralipid per mouse over 1 h) of propofol (AstraZeneca, UK) via tail vein in nude mice (6‐week‐old female BALB/c nude mice). The doses of 20 or 240 mg kg^−1^ of propofol were chosen according to the methods described in previous studies with modifications.^[^ ^44^ ^]^ Specifically, for mice in the low‐dose propofol group, first, 1.5 million HCT116 cells and then (with 1 min apart) 20 mg kg^−1^ propofol were injected. The same procedure was repeated for mice in the standard‐dose propofol group, but, 3 min after the administration of 20 mg kg^−1^ propofol, an angel catheter (size 26G, ZiBo Eastmed Healthcare Products Co., Ltd., Zibo, China) was placed in the mouse tail vein. Then, a syringe pump (WH‐SP‐08, Wenhao Mircofluidic Technology Co., Ltd., Suzhou, China) was used to inject propofol (240 mg kg^−1^) slowly (over 1 h). Four weeks after the injection of HCT116 cells and propofol, lung metastasis of tumor cells was detected by using ex vivo bioluminescence imaging. Tumor cells were injected before the injection of propofol to enable cells to be sufficiently exposed to propofol before propofol, which has half‐life of a 2 to 4 min,^[^ ^45^ ^]^ redistributed from blood into fat tissues. Note that low‐dose propofol could not be administered over 1 h because the mouse would move around under the low‐dose propofol. Thus, a single injection of low‐dose propofol was performed in the mouse.

### Injection of Propofol‐, Muscimol‐ and PP2‐Treated Tumor Cells in Mice

HCT116 cells overexpressing luciferase were treated with propofol (Sigma‐Aldrich, St. Louis, MO) (4 µg mL^−1^, the clinically relevant blood concentration of propofol) or DMSO (vehicle control) (Sigma‐Aldrich, St. Louis, MO) for 3 h in culture dishes. Specifically, propofol (4 µg mL^−1^) or DMSO (8 µL as vehicle control of propofol) was mixed with 6 million HCT116 cells in 8 mL cell culture medium for 3 h. HCT116 cells were then harvested and 1.5 million of propofol‐treated or DMSO‐treated HCT116 cells were injected through tail vein of 6‐week‐old female BALB/c nude mice. Tumor cell lung metastasis was detected in the two groups (DMSO vs propofol group) of mice via ex vivo bioluminescence imaging 4 weeks after injection. HCT116 cells overexpressing luciferase (6 million in 8 mL cell culture medium) were also treated with muscimol (Sigma‐Aldrich) (250 × 10^−6^ m) or water (20 µL as vehicle control of muscimol) for 3 h. The cultured cells were then harvested and 1.5 million cells were injected into the mouse via tail vein. In the PP2 intervention studies, mice were assigned to the following groups: 1) propofol (4 µg mL^−1^), 2) propofol (4 µg mL^−1^) plus PP2 (10 × 10^−6^ m) (Selleck), 3) muscimol (250 × 10^−6^ m), or 4) muscimol (250 × 10^−6^ m) plus PP2 (10 × 10^−6^ m). Six or more mice were included in each group in these studies.

### Bioluminescence Imaging

Bioluminescence imaging of mouse lungs was performed 4 weeks after intravenous injection of the tumor cells. A total of 150 mg kg^−1^ d‐luciferin solution (Yeasen, Shanghai, China) was injected intraperitoneally in the mice under brief isoflurane anesthesia. 15 min after luciferin injection, the mice (*N* = 6 per group) were euthanized to harvest whole lungs for ex vivo bioluminescent imaging to evaluate lung metastasis with the BLT AniView100 multimodal animal imaging system (Biolight Biotechnology, Guangzhou, China).

### Hematoxylin and Eosin (H&E) Staining

Harvested lungs were rinsed in PBS and fixed in 4% paraformaldehyde (Servicebio, Wuhan, China) overnight. Fixed lungs were embedded in paraffin, and sampled sections were taken across each lobe of lungs. Consecutive tissue (3 µm apart) sections were stained using H&E. To evaluate tumor metastasis, the total number of tumor nodules in all lung lobes per mouse (*N* = 6 to 9 per group) was counted on H&E‐stained sections using a microscope with sample identities disguised. Specifically, one section with the biggest diameter was obtained from each lobe of the lungs and the number of nodules in the section was counted for the quantification of tumor metastasis.

### Cell Culture

Human colorectal tumor cell lines HCT116 were cultured in McCoy's 5A medium (Gibco) with 10% fetal bovine serum (FBS) (Lonsera, S711‐001S, Uruguay). HUVEC line, human nonsmall‐cell lung carcinoma cell (NSCLC) line A549, and mouse colorectal tumor cell line CT26 were cultured in RPMI‐1640 medium (Gibco) with 10% FBS. Endometrial carcinoma cell line Ishikawa was cultured in DMEM/F12 medium (Gibco) with 10% FBS. The 293FT cell line was cultured in DMEM medium (Gibco) with 10% FBS. Cells were cultured at 37 °C, 5% CO_2_ atmosphere. Breast tumor cell line MDA‐MB‐231 was cultured in Leibovitz's L15 medium (Gibco) at 37 °C. All cells tested negative for mycoplasma. HCT116, CT26, and MDA‐MB‐231 cell lines were purchased from the cell bank of the Chinese Academy of Sciences. A549 and Ishikawa cell lines were gifts from Dr. Jiuhong Kang at Tongji University. HUVEC was a gift from Dr. Jialin Charles Zheng's lab at Tongji University. The 293FT cell line was a gift from Dr. Xiaoqing Zhang's lab at Tongji University.

### Tumor Cell–HUVEC Adhesion Assay

1.5 × 10^5^ HUVECs per well were seeded in 24‐well plates overnight for cell monolayer formation. A total of 5 × 10^4^ tumor cells stained with Calcein AM (Yeasen) were seeded on HUVECs per well. Cells were incubated to assess the interaction of tumor cells with HUVECs. Because different tumor cell lines have different adhesion capabilities, the incubation time differed for each cell line: CT26 cells, 2 h; HCT116 cells, 6 h; MDA‐MB‐231 cells, 3 h; A549 cells, 2 h; and Ishikawa cells, 5 h. After incubation, cells were washed to remove nonadherent cells. Images of cells were captured using a fluorescence inverted microscope with three different visual fields per dish (six dishes per group) with sample identities disguised.

### Membrane Potentiometric Analysis of HCT116 Cells

Voltage‐sensitive dye bis‐(1,3‐dibutylbarbituric acid) trimethine oxonol [DiBAC4(3)]^[^ ^46^ ^]^ (Sigma‐Aldrich) (final concentration of 20 × 10^−6^ m) and *γ*‐GABA (Selleck) (final concentration of 250 × 10^−6^ m) were added to HCT116 culture medium without FBS, and cells were incubated for 10 min. Green fluorescence of cells were captured using a fluorescence inverted microscope (Zeiss observer Z1, Germany). Cells were then incubated with bicuculine (Selleck) (200 × 10^−6^ m) or DMSO for 5 min and green fluorescence of cells was assessed. The experiment was repeated six times, and three fields were viewed per sample. The intensity of the fluorescence in each sample was determined by using ImageJ (version 8.0, NIH, Bethesda, MD).

### Src siRNA

The sequence of siRNA for Src knockdown was accessed from a previous study.^[^ ^47^ ^]^ The most effective downregulating sequences of siRNA were chosen as: PF: 5″‐CAAGAGCAAGCCCAAGGAUtt‐3″; PR: 5″‐AUCCUUGGGCUUGCUCUUGtt‐3″. The control siRNA sequence was: PF: 5″‐UUCUCCGAACGUGUCACGUtt‐3″; PR: 5″‐ACGUGACACGUUCGGAGAAtt‐3″.

### Plasmids

For knockdown of TRIM21, double‐stranded oligo shRNA sequences were cloned into lentiviral vector pLKO.1. The primer sequences^[^ ^48^ ^]^ were: TRIM21 shRNA‐1, PF: 5′‐CCGGTGGAAGTGGAAATTGCAATAACTCGAGTTATTGCAATTTCCACTTCCATTTTTG‐3′, PR: 5′‐AATTCAAAAATGGAAGTGGAAATTGCAATAACTCGAGTTATTGCAATTTCCACTTCCA‐3′; TRIM21 shRNA‐2, PF: 5′‐CCGGCAATCCGTGGCTGATACTTTCCTCGAGGAAAGTATCAGCCACGGATTGTTTTTG‐3′, PR: 5′‐AATTCAAAAACAATCCGTGGCTGATACTTTCCTCGAGGAAAGTATCAGCCACGGATTG‐3′; shRNA control vector primer sequences, PF: 5′‐CCGGCCTAAGGTTAAGTCGCCCTCGCTCGAGCGAGGGCGACTTAACCTTAGGTTTTTG‐3′, PR: 5′‐AATTCAAAAACCTAAGGTTAAGTCGCCCTCGCTCGAGCGAGGGCGACTTAACCTTAGG‐3′. Virus was packaged by 293FT cells and used to infect HCT116 cells. pCMV3‐TRIM21 with C‐HA (HG18010‐CY, Sino Biological, Beijing, China) or pCMV3‐TRIM21 with C‐Myc (HG18010‐CM, Sino Biological) was used for human TRIM21 overexpression. pCMV3‐Src with C‐Flag (HG29841‐CF, Sino Biological) was used for human Src overexpression. Plasmids with overexpression of Src or TRIM21 were transiently transfected into cells by using X‐tremeGENE 9 DNA transfection reagent (Roche, Switzerland) as per manufacturer's recommended protocol.

### KO of GABA_A_R Subunit *β*3 (GABRB3) and *δ* (GABRD) in HCT116 Cells

A vector with clustered regularly interspaced short palindromic repeats (CRISPR)/Cas9‐mediated KO of GABA_A_R subunit *β*3 was designed, which was synthesized by Genomeditech, Ltd. (Shanghai, China). The sequence of gRNA was: Primer‐control‐T: 5′‐caccgACGGAGGCTAAGCGTCGCAA‐3′, Primer‐control‐B: 5′‐aaacTTGCGACGCTTAGCCTCCGTc‐3′; GABRB3 sgRNA Primer‐T: 5′‐CACCGATAAAAGGCTCGCCTATTCT‐3′, Primer‐B: 5′‐AAACAGAATAGGCGAGCCTTTTATC‐3′. gRNA was inserted into the LentiGuid‐EF1a‐Neo (stuffer) vector. Then, the virus was packaged, and HCT116 cells were infected with the cas9 overexpression virus. Stable cell lines were screened by blasticidin. Finally, cas9 HCT116 cells were infected by gRNAs lentivirus to induce the knockout of GABRB3 screened by Neomycin. Then, a vector with CRISPR/Cas9‐mediated KO of GABRD was further designed, which was also synthesized by Genomeditech, Ltd. The sequence of gRNA was: Primer‐control‐T: 5′‐caccgACGGAGGCTAAGCGTCGCAA‐3′, Primer‐control‐B: 5′‐aaacTTGCGACGCTTAGCCTCCGTc‐3′; GABRD sgRNA Primer‐T1: 5′‐CACCGCGATGCCAGGCCGGAAGTTG‐3′, Primer‐B: 5′‐AAACCAACTTCCGGCCTGGCATCGC‐3′. The gRNA was inserted into the LentiGuid‐EF1a‐Puro vector. The virus was then packaged to infect the previously constructed control and GABRB3 KO HCT116 cells, respectively. Stable cell lines were screened by puromycine to complete the construction of HCT116 cells with KO of both GABRB3 and GABRD.

### Western Blot (WB)

Cells were lysed using lysis buffer (M‐PER Mammalian Protein Extraction Reagent, Thermo, USA) with protease inhibitor cocktail (Bimake, Shanghai, China). Protein was transferred onto polyvinylidene fluoride membranes (Bio‐Rad, CA, USA). Primary antibodies included anti‐GAPDH (AP0063, 1:1000 dilution; Bioworld, Nanjing, China), anti‐TRIM21 (12108‐1‐AP, 1:1000 dilution; Proteintech Group, Chicago, IL, USA), anti‐Phospho‐Src (Tyr416) (p‐Src) (2101S, 1:1000 dilution; Cell Signaling Technology, Danvers, MA, USA), anti‐Src (2109T, 1:1000 dilution; Cell Signaling Technology), and anti‐GABA_A_R *β*3 (GABRB3) (ab98968, 1:1000 dilution; Abcam, UK), anti‐GABA_A_R *δ* (GABRD) (abs141150, 1:1000 dilution; Absin Bioscience Inc., Shanghai, China), anti‐FAK (sc‐271195, 1:1000 dilution; Santa Cruz Biotechnology, Santa Cruz, CA), anti‐Phospho‐FAK (Tyr397) (p‐FAK) (611807, 1:1000 dilution; BD Transduction Laboratories, San Jose, CA), anti‐HA‐tag (M180‐3, 1:1000 dilution; MBL, Japan), anti‐Myc‐tag (2276S, 1:1000 dilution; Cell Signaling Technology), and anti‐Flag‐tag (14793S, DYKDDDDK Tag, 1:1000 dilution; Cell Signaling Technology). Secondary antibodies were antirabbit IgG horseradish peroxidase (HRP)‐linked antibody (7074S, 1:3000 dilution; Cell Signaling Technology) and antimouse IgG HRP‐linked antibody (7076S, 1:3000 dilution; Cell Signaling Technology). ChemiDoc XRS+ system (Bio‐Rad) was used to detect protein signal by enhanced chemiluminescence (Clarity Western ECL substrate, Bio‐Rad). To detect total Src and FAK proteins, the blots were stripped with WB stripping buffer (Thermo Fisher Scientific, Waltham, MA, USA) after detection of the p‐Src and p‐FAK proteins, and reprobed with Src and FAK antibodies.

### Src Ubiquitylation Assay

For analyzing the effects of GABA_A_R or propofol on regulating Src ubiquitylation, Src‐flag and ubiquitin‐HA vectors were transfected into HCT116 cells using X‐tremeGENE 9 DNA transfection reagent (Roche). 24 h later, cells were treated with muscimol, bicuculine, or propofol for 3 h. Protein lysates were obtained from the cells lysed by lysis buffer (Pierce IP lysis buffer, Thermo) and incubated with anti‐flag gel beads (EZview Red Anti‐flag Affinity Gel, Sigma) at 4 °C overnight. Then, beads were washed three times with lysis buffer. The proteins were released from the beads for western blot and analyzed by anti‐HA antibody (MBL) to evaluate the ubiquitylation. For analyzing the TRIM21 regulating Src ubiquitylation, TRIM21 overexpression vector or control vector was transfected into HCT116 cells with Src‐flag and ubiquitin‐HA vector. Then, the experiment was repeated to assess Src ubiquitylation.

### Co‐Immunoprecipitation (Co‐IP)

Co‐IP was used to detect whether there was a direct interaction of TRIM21 and Src. The TRIM21‐C‐Myc and Src‐Flag overexpressing vectors were transfected into HCT116 cells by using X‐tremeGENE 9 DNA transfection reagent (Roche, Switzerland). 48 h later, cells were lysed by lysis buffer (Pierce TM IP lysis buffer, Thermo) and incubated with anti‐Myc gel beads (EZview Red Anti‐C‐Myc Affinity Gel, Sigma) at 4 °C overnight. Then, beads were washed three times with lysis buffer. The proteins were released from the beads for western blot and examined by using the TRIM21 antibody (12108‐1‐AP, Proteintech Group).

### CHX Chase Assay

HCT116 cells were treated with muscimol, propofol, or control condition for 3 h. Then, the medium was replaced and CHX (final concentration 50 µg mL^−1^) (Selleck) was added for indicated times (0, 4, 8, or 12 h). Finally, cells were lysed for western blot to detect Src protein.

### Immunofluorescence Staining

Cover glass was treated with 0.1 mg mL^−1^ polylysine (Beyotime, Shanghai, China). After drying in air, laminin (10 ng mL^−1^) (Thermo) was added on the cover glass and it was dried, which simulated the HUVEC extracellular matrix for adhesion with tumor cells. Cells were seeded onto this coated cover glass for adhesion and extension studies and then were fixed in 4% paraformaldehyde in PBS (pH 7.4) for 20 min. Cell membranes were permeated with 0.2% Triton X‐100 in PBS for 8 min. Cells were blocked for 1 h in 2% BSA in PBS. Then, cells were incubated with primary antibodies: anti‐Vinculin (ab129002, 1:100 dilution; Abcam, UK), anti‐Talin (ab11188, 1:100 dilution; Abcam), and ‐TRIM21 (12108‐1‐AP, 1:100 dilution; Proteintech Group). Secondary antibodies were antirabbit IgG (H+L) F(ab′)2 Fragment (Alexa Fluor 568, 8889S, 1:1000 dilution; Cell Signaling Technology) and antimouse IgG (H+L) F(ab′)2 Fragment (Alexa Fluor 488,4408S, 1:1000 dilution; Cell Signaling Technology). Imaging was performed with a confocal microscope (Olympus, FV3000, Japan).

### Quantitative Real‐Time PCR (qRT‐PCR)

Total RNA of HCT116 cells was isolated using RNAiso Plus (Takara, Dalian, China). 400 ng of RNA was used to synthesize cDNA with a reverse‐transcription PCR kit (Takara). qRT‐PCR primer sequences were as follows: *Src* PF, 5′‐TTCCACGGCAAGATCACACG‐3′, PR, 5′‐GCATGGTACATGATGCGGTAG‐3′; GAPDH PF, 5′‐ACCACAGTCCATGCCATCAC‐3′, PR, 5′‐TCCACCACCCTGTTGCTGTA‐3′; *TRIM21* PF, 5′‐TGCTGCAGGAGGTGATAAT‐3′, PR, 5′‐TCCTGAGTTCTGGAGAGGTAATA‐3′.

### Statistical Analysis

The ex vivo bioluminescence imaging data was log‐normalized prior to analysis. Total sample size (*N*) was given for each experiment as follows: ex vivo bioluminescence imaging (*N* = 6 to 10); H&E staining (*N* = 6 to 9). Three independent repeat qRT‐PCR experiments were conducted with each group. Data from ex vivo bioluminescence imaging and qRT‐PCR were presented as mean ± standard deviation (SD). Histological analysis data were presented using median and interquartile range. Student's *t*‐test was used to determine differences in ex vivo lung bioluminescence among mice in control, propofol, muscimol, propofol plus PP2, and muscimol plus PP2 groups. The Mann–Whitney *U* test was used to determine differences in numbers of metastatic nodules in the lungs of mice in the same group. Student's *t*‐test and one‐way ANOVA test were used to determine differences in mRNA expression between different treatments. Student's *t*‐test and one‐way ANOVA test with post‐hoc Bonferroni test were used to determine the quantification of the HCT116 cells adhesion to an HUVECs monolayer. One‐way ANOVA test with post‐hoc Bonferroni test was used to determine the quantification of the membrane‐localized Talin and Vinculin in focal adhesion. In all the cases, *P*‐values <0.05 were considered statistically significant, and significance testing was two‐tailed. Asterisks and pound sign indicate the level of significance as follows: * and ^#^, ** and ^##^, or *** and ^###^ for the *P*‐values <0.05, <0.01, or <0.001, respectively. Statistical analysis was carried out using GraphPad Prism software (version 8.0; GraphPad Software, La Jolla, CA, USA).

## Conflict of Interest

The authors declare no conflict of interest of the present study. Dr. Zhongcong Xie provides consulting service to Baxter, Shanghai 9th and 10th Hospital.

## Author Contributions

Q.L., Z.S., C.C., and H.Z. contributed equally to this work. Q.L., Z.S., C.C., and H.Z. conceived and carried out the experiment and analyzed the data. Y.S. and Z.X. jointly supervised the project and drafted the manuscript with input from all authors. M.L., R.L., and P.W. provided advice to the studies and critical comments to the manuscript.

## Supporting information

Supporting Information

## Data Availability

Data of this study will be provided from the corresponding authors upon reasonable request.
