# Supplementary material for: Anesthetic Propofol Promotes Tumor Metastasis in Lungs via GABAAR‐Dependent TRIM21 Modulation of Src Expression
Source: Adv Sci (Weinh). 2021 Jul 15;8(18):2102079. doi: 10.1002/advs.202102079 (PMC8456212; doi:10.1002/advs.202102079)
Supplement: Supplementary file 1 — Supporting Information [file ADVS-8-2102079-s001.pdf]

© 2021 Wiley-VCH GmbH

ADVANCED SCIENCE
Open Access

# Supporting Information

for *Adv. Sci.*, DOI: 10.1002/advs.202102079

# Anesthetic propofol promotes tumor metastasis in lungs via $GABA_A$R-dependent TRIM21 modulation of Src expression

*Qidong Liu, Zhihao Sheng, Chun Cheng, Hui Zheng, Michael Lanuti, Rong Liu, Ping Wang, Yuan Shen* and Zhongcong Xie**

**Supporting Information**

# Anesthetic propofol promotes tumor metastasis in lungs via $GABA_AR$-dependent TRIM21 modulation of Src expression

Qidong Liu, Zhihao Sheng, Chun Cheng, Hui Zheng,
Michael Lanuti, Rong Liu, Ping Wang, Yuan Shen and Zhongcong Xie

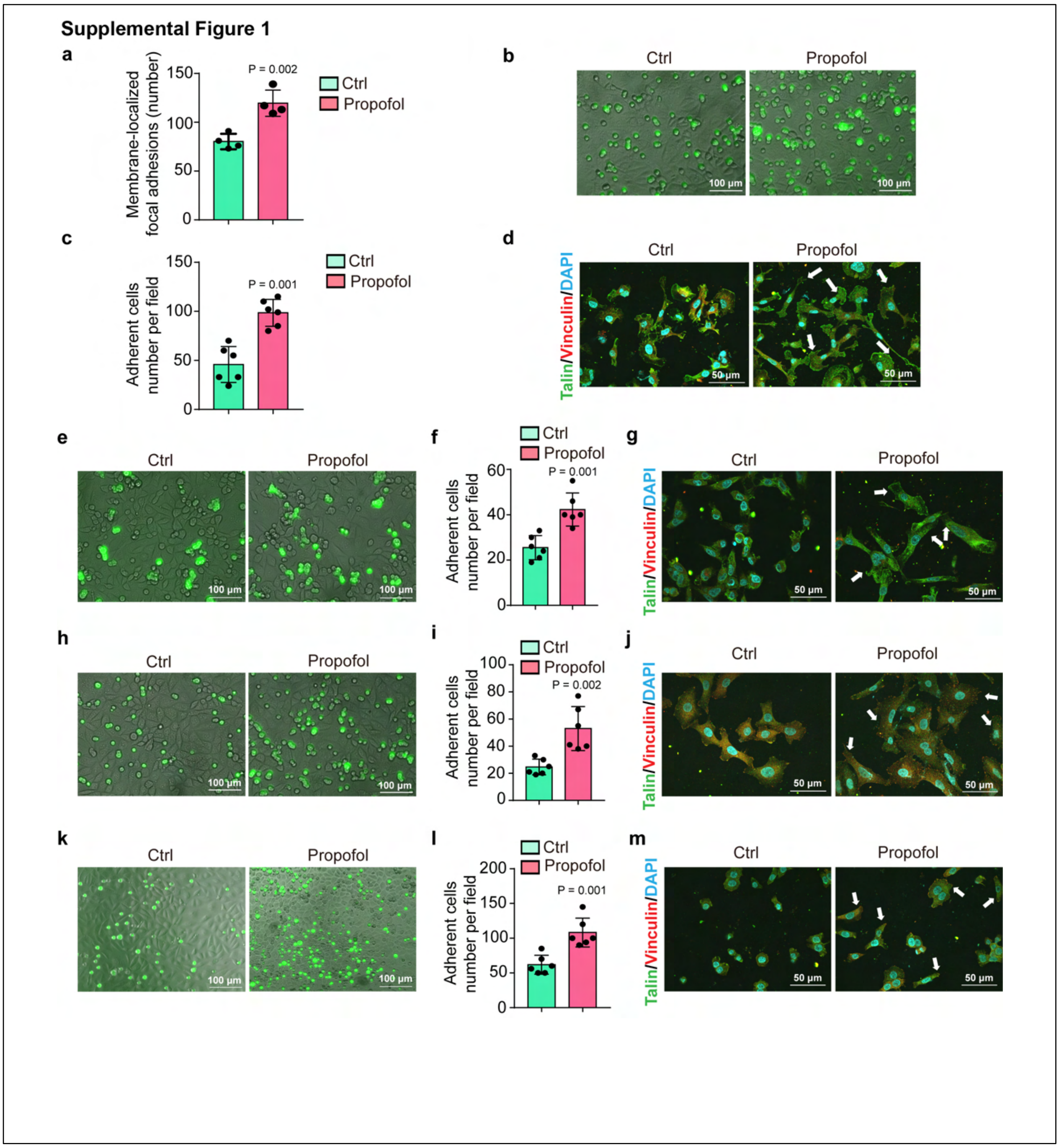


**Supplemental Figure 1. Propofol enhances adhesion and extension of tumor cells.**

**a.** Quantification of the membrane-localized Talin and Vinculin in focal adhesions in HCT116 cells treated with propofol or control condition (N = 4, mean ± SD, Student's t-test, P = 0.002), Ctrl, control. Fluorescent micrographs and immunofluorescent staining of Talin and Vinculin show adhesion of HUVEC monolayer with the propofol-treated CT26 (**b, c, d**), MDA-MB-231 (**e, f, g**), A549 (**h, i, j**), Ishkawa (**k, l, m**) and control cells (scale bar = 50 μm or 100 μm). Arrows indicate focal adhesions. Ctrl, control; HUVEC, human umbilical vein endothelial cell; SD, standard deviation.

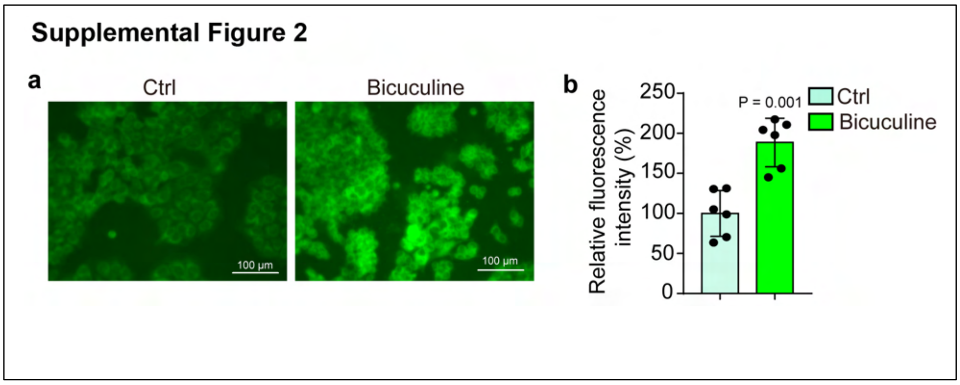


**Supplemental Figure 2. GABA$_A$R antagonist bicuculine induces depolarization of membrane potential in HCT116 cells.**

**a**. Fluorescent micrographs of DiBAC4(3)-dyed HCT116 cells treated with control condition or GABA$_A$R antagonist (bicuculine, 250 µM) (scale bar = 100 µm). **b**. The relative quantification of fluorescence intensity was determined (N = 6, mean ± SD, Student's t-test, P = 0.001). Ctrl, control; SD, standard deviation.

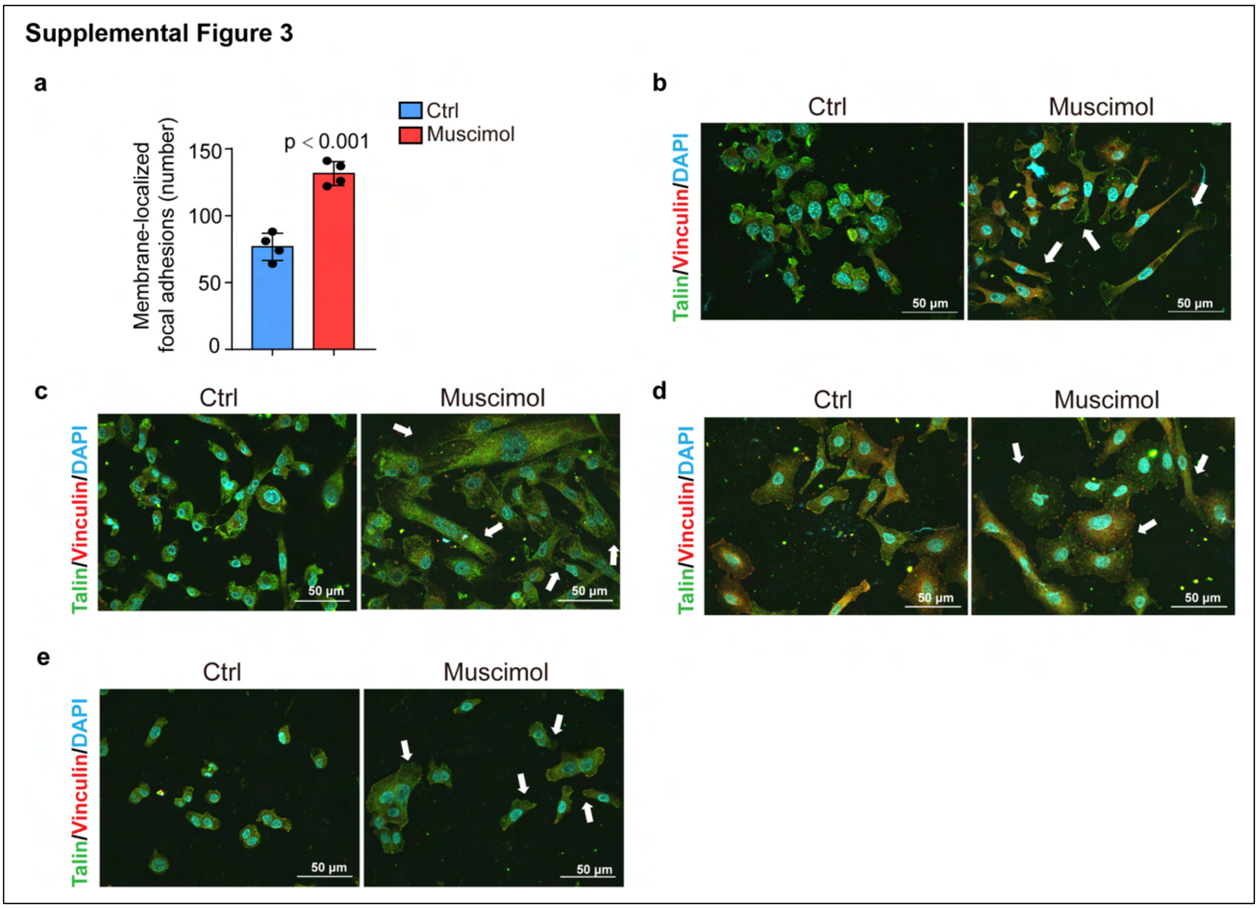


**Supplemental Figure 3. GABA$_A$R agonist muscimol promotes tumor cells adhesion and extension.**

**a.** Quantification of the membrane-localized Talin and Vinculin in focal adhesions in HCT116 cells treated with muscimol or control (N = 4, mean ± SD, Student's t-test, $P < 0.001$). Immunofluorescence staining of Talin and Vinculin in muscimol-treated CT26 (**b**), MDA-MB-231 (**c**), A549 (**d**), and Ishkawa (**e**) cells compared to controls (scale bar = 50 µm). Arrows indicate focal adhesions. Ctrl, control; SD, standard deviation.

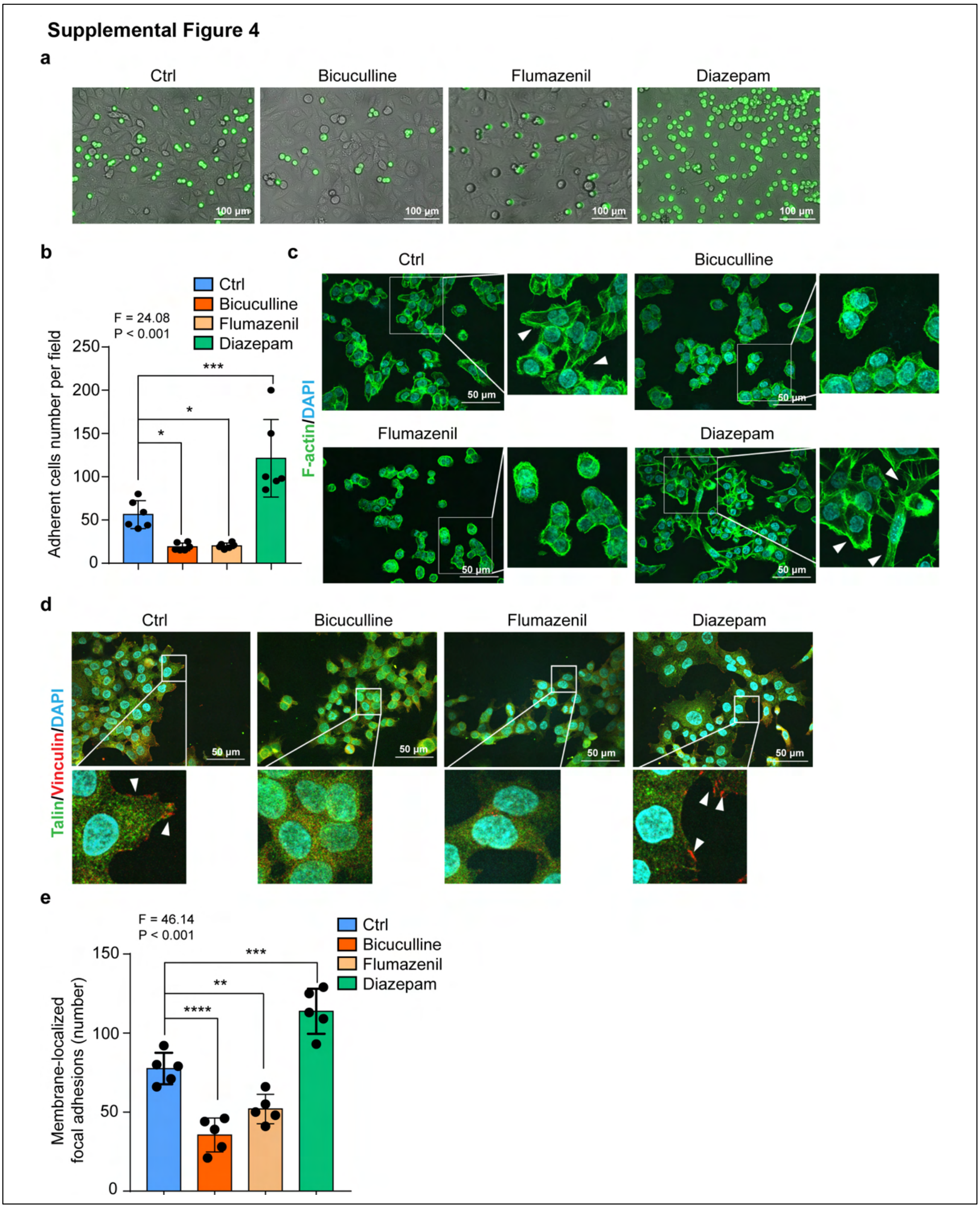


**Supplemental Figure 4. GABA$_A$R contributes to the regulation of HCT116 cells adhesion and extension.**

**a**. Fluorescent micrographs showing adhesion between the HUVEC monolayer and the HCT116 cells (green) treated with GABA$_A$R antagonist bicuculine, benzodiazepine antagonist flumazenil, or benzodiazepine agonist diazepam (scale bar = 100 μm). **b.** Quantification of the

adhesion between the HUVEC monolayer and the HCT116 cells under static conditions in each group (N = 6, mean ± SD, One-way ANOVA test with post-hoc Bonferroni test, F = 24.08, P < 0.001). **c**. F-actin labeling of membrane protrusions in HCT116 cells treated with bicuculine, flumazenil, or diazepam compared to controls (F-actin: phalloidin; nucleus: DAPI; scale bar = 50 μm). Arrows indicate extensions of cell membrane protrusions. **d.** Immunofluorescence staining of Talin and Vinculin in HCT116 cells treated with bicuculine, flumazenil, or diazepam compared to controls. (scale bar = 50 μm). Arrows indicate focal adhesions. Ctrl, control. **e.** Quantification of the membrane-localized Talin and Vinculin in focal adhesions in **d** (N = 5, mean ± SD, One-way ANOVA test with post-hoc Bonferroni test, F = 46.14, P < 0.001). Ctrl, control; HUVEC, human umbilical vein endothelial cell; SD, standard deviation. * P < 0.05, ** P < 0.01, *** P < 0.001, **** P < 0.0001.

## Supplemental Figure 5

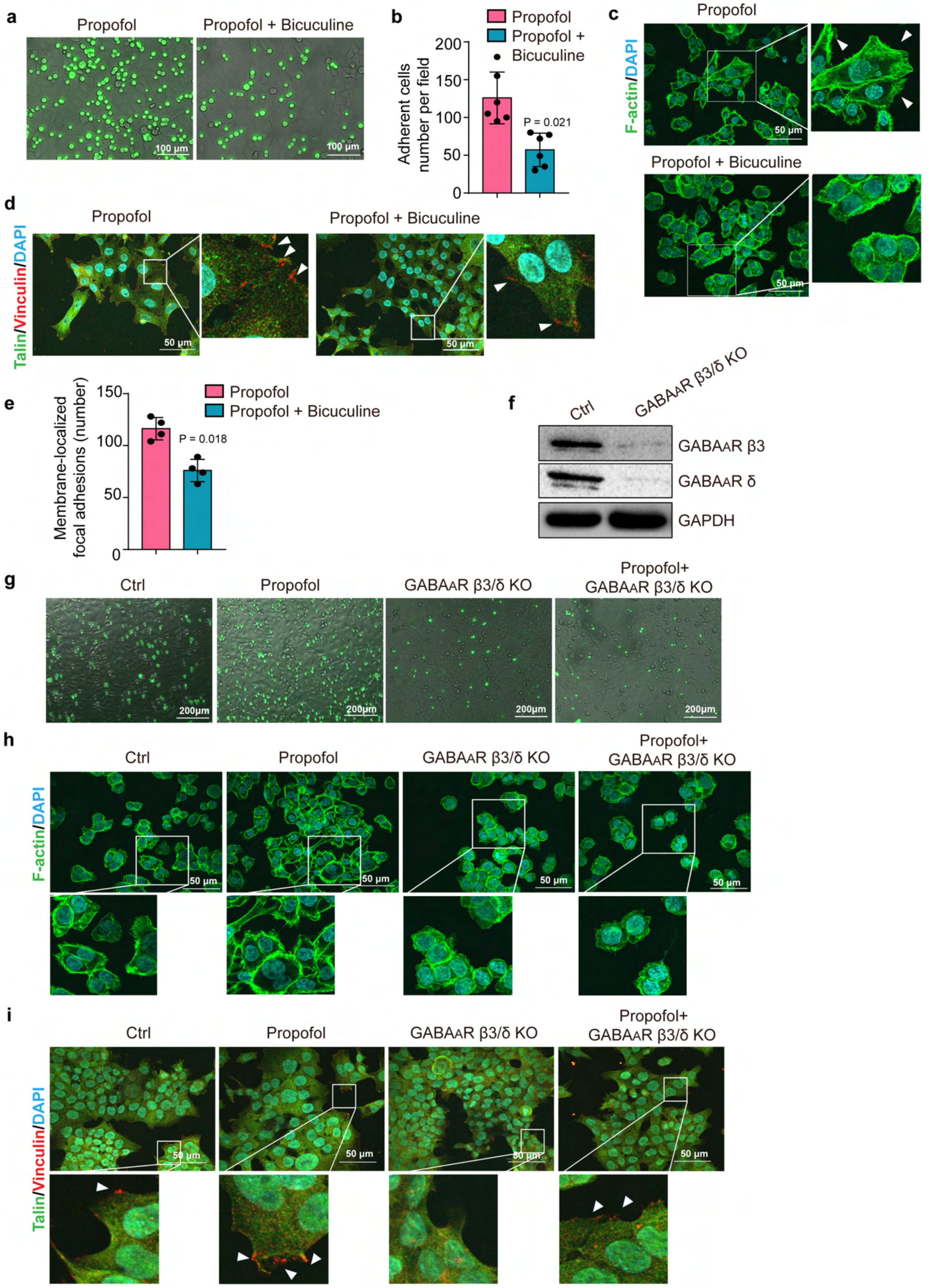

**Supplemental Figure 5. Inhibition of $GABA_AR$ attenuates the propofol-induced enhancement of tumor cells adhesion and extension.**

**a**. Fluorescent micrographs showing adhesion between the HUVEC monolayer and the propofol-treated HCT116 cells (green) with or without $GABA_AR$ antagonist bicuculine (scale bar = 100 μm). **b**. Quantification of the adhesion between HCT116 cells and HUVEC monolayer (N = 6, mean ± SD, Student's t-test, P = 0.021). **c.** F-actin labeling of membrane protrusions in propofol-treated HCT116 cells with or without $GABA_AR$ antagonist bicuculine (scale bar = 100 μm). Arrows indicate extensions of cell membrane protrusions. **d**. Immunofluorescence staining of Talin and Vinculin in the propofol-treated HCT116 cells with or without $GABA_AR$ antagonist bicuculine (scale bar = 50 μm). Arrows indicate focal adhesions. Ctrl, control. **e.** Quantification of the membrane-localized Talin and Vinculin in focal adhesions in (d) (N = 4, mean ± SD, Student's t-test, P = 0.018). **f**. Western blot showing the knockout of both of $GABA_AR$ subunit β3 and δ. **g**. Fluorescent micrographs showed that knockout of $GABA_AR$ blocked the adhesion of HCT116 cells (green) to the HUVEC monolayer (scale bar = 200 μm). **h**. F-actin labeling of membrane protrusions (scale bar = 50 μm). **i**. Immunofluorescence staining of Talin and Vinculin of focal adhesions (scale bar = 50 μm). Arrows indicate focal adhesions. Ctrl, control; HUVEC, human umbilical vein endothelial cell; SD, standard deviation.

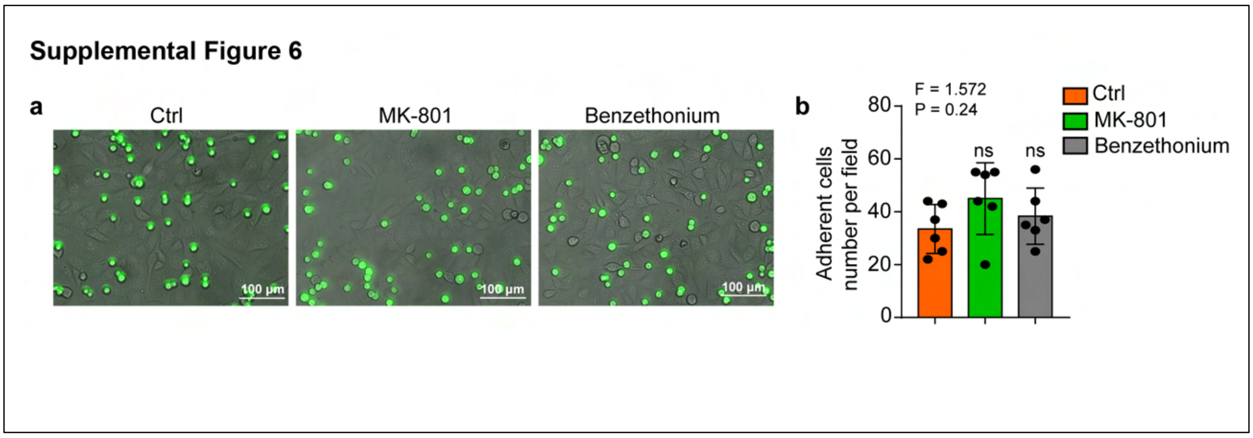


**Supplemental Figure 6. Inhibition of NMDA receptor or acetylcholine receptor does not regulate cell adhesion.**

**a.** Fluorescent micrographs showing adhesion between the HUVEC monolayer and the HCT116 cells (green) treated with control condition, NMDA receptor antagonist MK-801 (500 μM) or acetylcholine receptor antagonist benzethonium (10 μM) (scale bar = 100 μm). **b**. Quantification of the adhesion between HCT116 cells and HUVEC monolayer (N = 6, mean ± SD, One-way ANOVA test with post-hoc Bonferroni test, F = 1.572, P = 0.24). Ctrl, control; HUVEC, human umbilical vein endothelial cell.

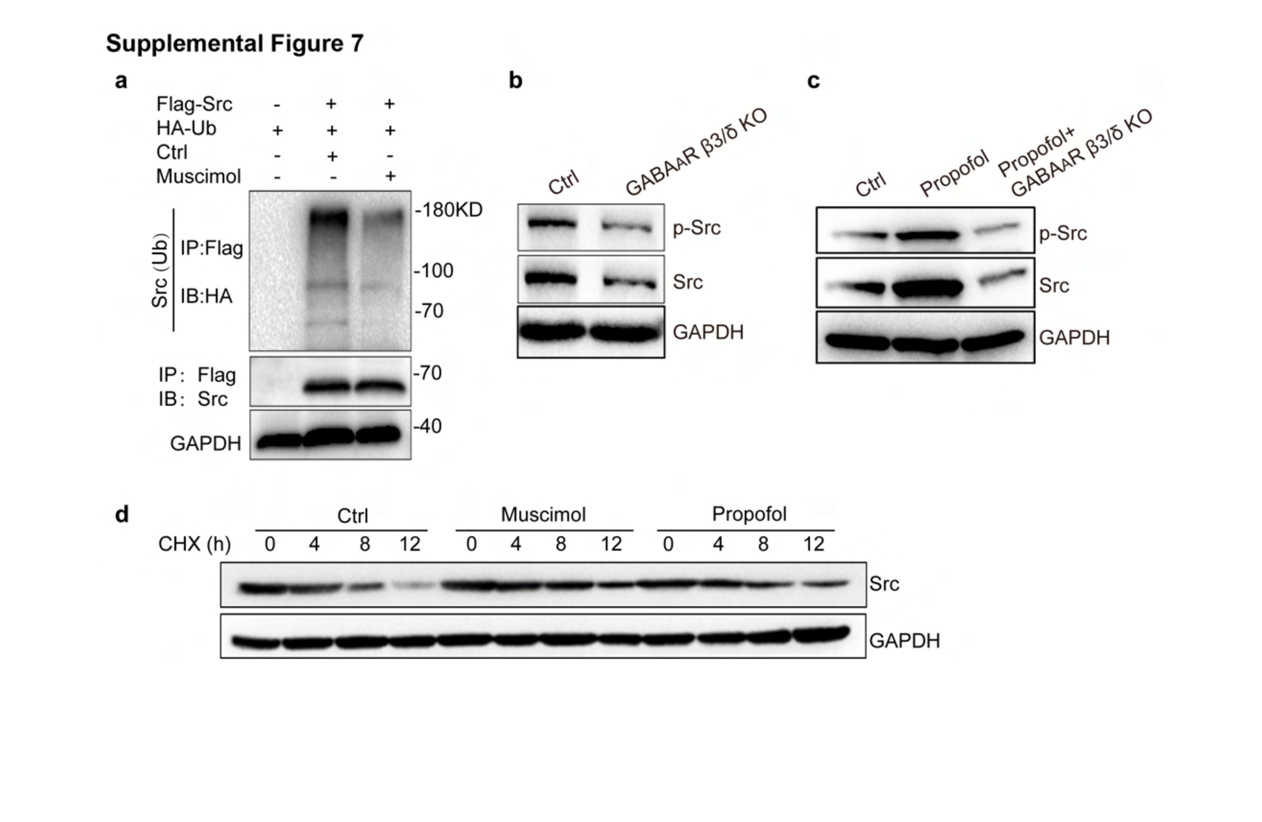


**Supplemental Figure 7. GABA$_A$R mediates the propofol regulating expression of Src.**

**a.** Western blot analysis of Src ubiquitylation in HCT116 cells following overexpression of Src-Flag or control vector and treatment with muscimol or control condition. Src was immunoprecipitated with anti-Flag and immunoblotted with anti-HA. **b.** Western blot analysis of Src and p-Src expression in HCT116 cells following knockout of both GABA$_A$R subunit β3 and δ. **c.** Western blot analysis of Src and p-Src expression in HCT116 cells (with or without knockout of the GABA$_A$R subunit β3 and δ) treated with propofol. GAPDH is a loading control. **d.** CHX chase assay indicated that the half-life of Src protein was markedly prolonged, by western blot analysis, with the treatment of muscimol or propofol as compared to control conditions. GAPDH is a loading control. Ctrl, control; p-Src, phosphorylated Src; CHX, Cycloheximide.

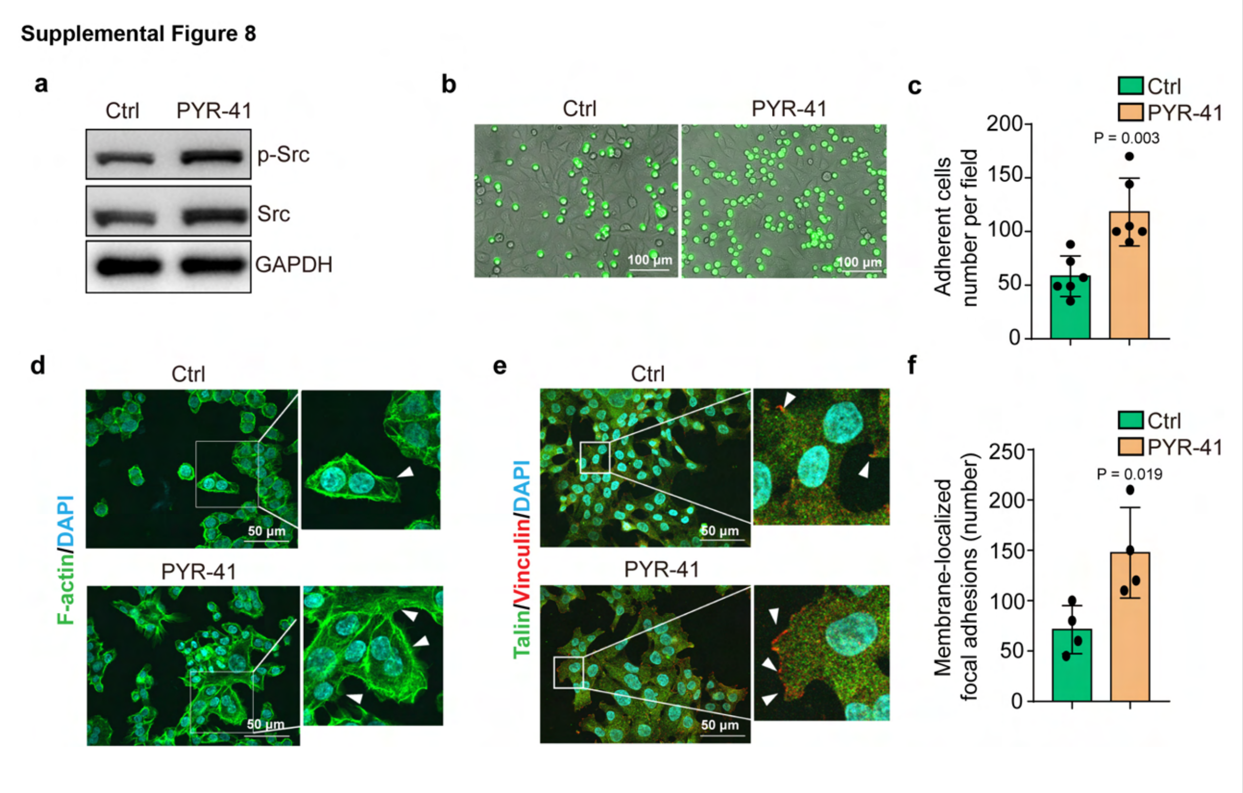


**Supplemental Figure 8. Inhibition of ubiquitylation upregulates Src expression and enhances HCT116 cells adhesion and extension.**

**a.** Western blot analysis of Src and p-Src expression in HCT116 cells treated with E1 enzyme inhibitor PYR-41 or control condition. GAPDH is a loading control. **b.** Fluorescent micrographs showing adhesion of HCT116 cells (green) treated with PYR-41 or control condition to the HUVEC monolayer (scale bar = 100 µm). **c**. Quantification of the adhesion between the HUVEC monolayer and the HCT116 cells (N = 6, mean ± SD, Student's t-test, P = 0.003). **d.** F-actin labeling of membrane protrusions in HCT116 cells treated with PYR-41 or control condition (scale bar = 50 µm). Arrows indicate extensions of cell membrane protrusions. **e.** Immunofluorescence staining of Talin and Vinculin in HCT116 cells treated with PYR-41 or control condition (scale bar = 50 µm). Arrows indicate focal adhesions. Ctrl, control. **f.** Quantification of the membrane-localized Talin and Vinculin in focal adhesions (N = 4, mean ± SD, Student's t-test, P = 0.019). Ctrl, control; p-Src, phosphorylated Src; HUVEC, human umbilical vein endothelial cell; SD, standard deviation.

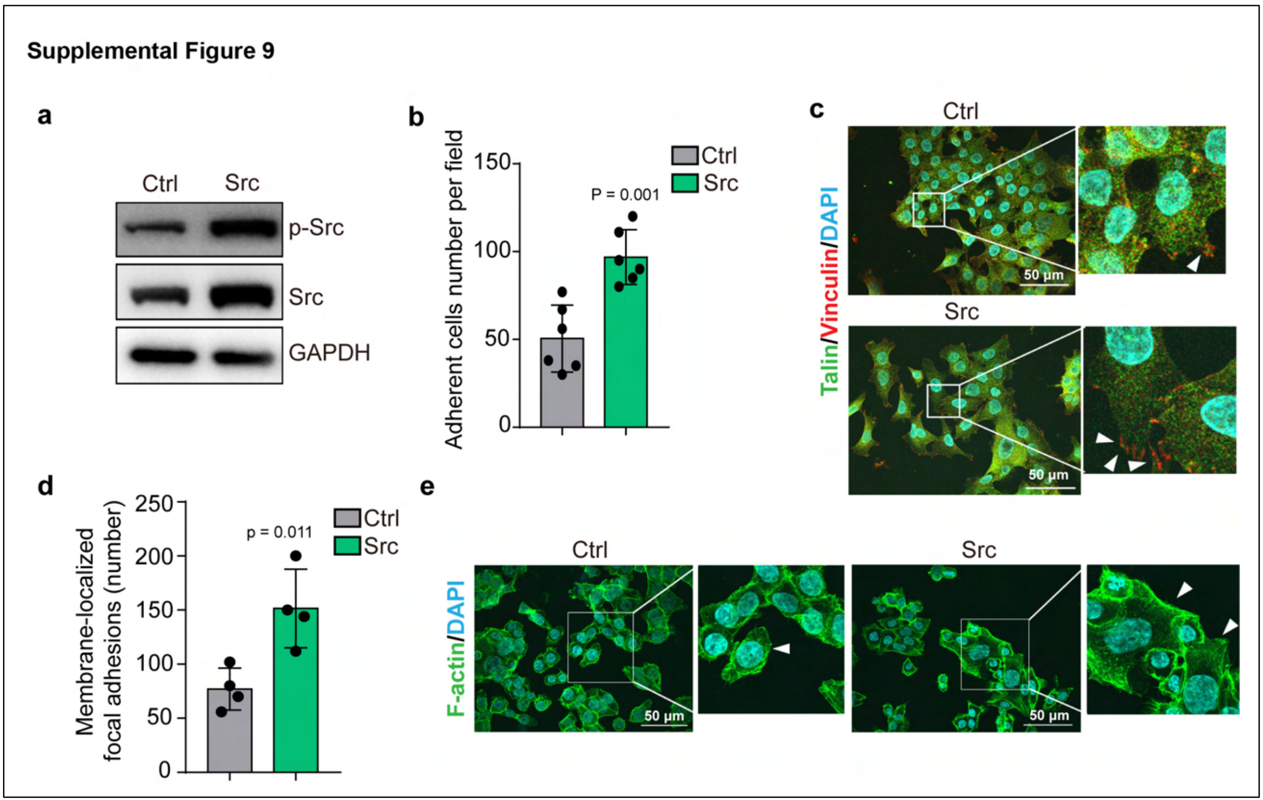


**Supplemental Figure 9. Overexpression of Src promotes HCT116 cell adhesion and extension.**

**a.** Western blot analysis of Src and p-Src expression in HCT116 cells with or without Src overexpression. GAPDH is a loading control. **b.** Quantification of the adhesion between HCT116 cells and HUVEC monolayer (N = 6, mean ± SD, Student's t-test, P = 0.001). **c.** Immunofluorescence staining of Talin and Vinculin in HCT116 cells with or without overexpression of Src (scale bar = 50 µm). Arrows indicate focal adhesions. Ctrl, control. **d.** Quantification of the membrane-localized Talin and Vinculin in focal adhesions (N = 4, mean ± SD, Student's t-test, P = 0.011). **e.** F-actin labeling of membrane protrusions in HCT116 cells with or without overexpression of Src (scale bar = 50 µm). Arrows indicate extensions of cell membrane protrusions. Ctrl, control; p-Src, phosphorylated Src; HUVEC, human umbilical vein endothelial cell; SD, standard deviation.

**Supplemental Figure 10**

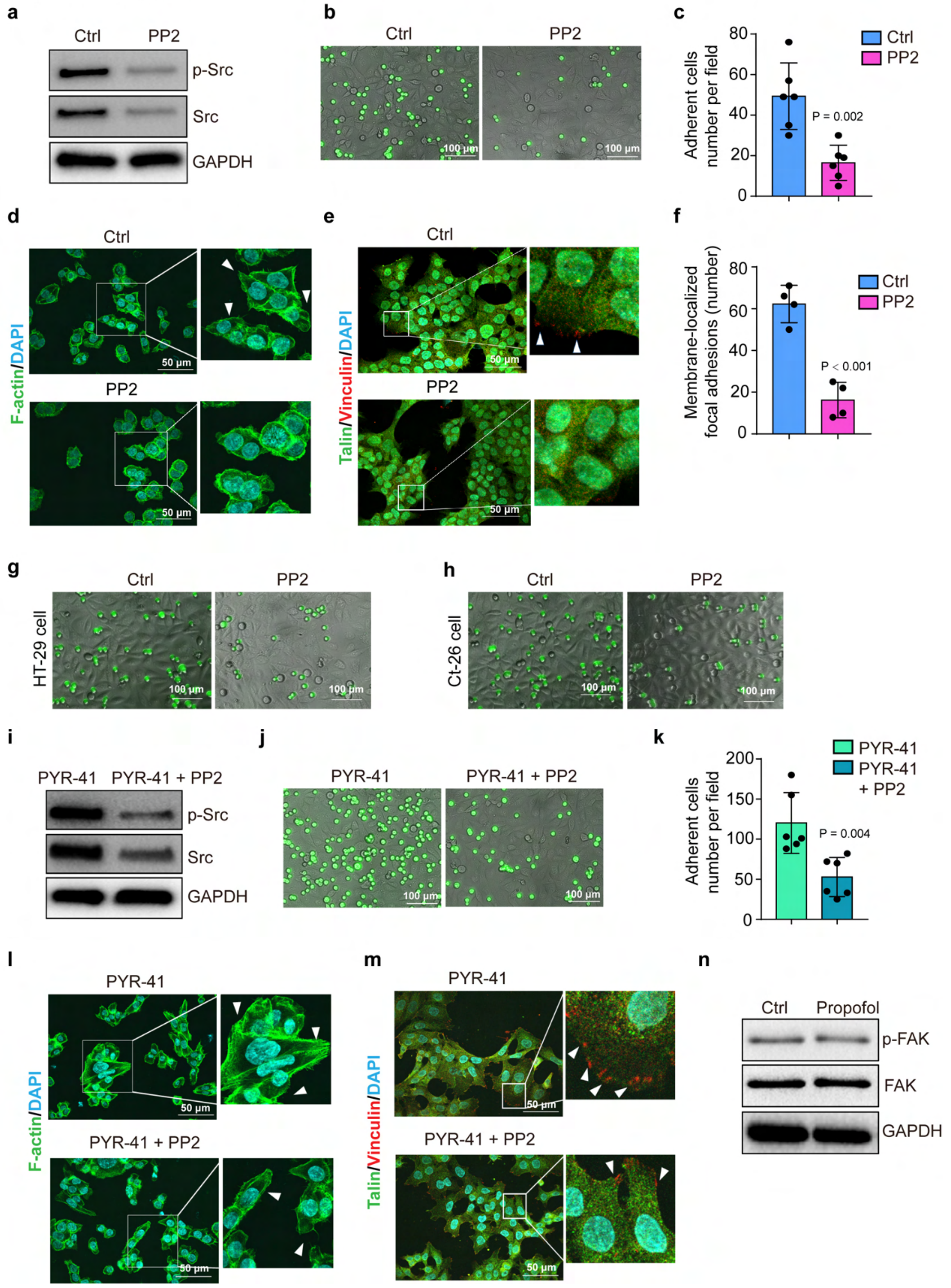

**Supplemental Figure 10. Inhibition of Src attenuates tumor cell adhesion and extension.**

**a.** Western blot analysis of Src and p-Src expression in HCT116 cells treated with Src inhibitor PP2. GAPDH is a loading control. **b.** Fluorescent micrographs demonstrating adhesion between the HUVEC monolayer and the HCT116 cells (green) treated with PP2 or control condition (scale bar = 100 μm). **c**. Quantification of the adhesion between HCT116 cells and HUVEC monolayer (N = 6, mean ± SD, Student's t-test, P = 0.002). **d.** F-actin labeling of membrane protrusions in HCT116 cells treated with PP2 or control condition (scale bar = 50 μm). Arrows indicate extensions of cell membrane protrusions. **e.** Immunofluorescence staining of Talin and Vinculin in HCT116 cells treated with PP2 or control condition (scale bar = 50 μm). Arrows indicate focal adhesions. Ctrl, control. **f.** Quantification of the membrane-localized Talin and Vinculin in focal adhesions (N = 4, mean ± SD, Student's t-test, P < 0.001). **g.** Fluorescent micrographs demonstrating adhesion between the HUVEC monolayer and the HT-29 cells (green) treated with PP2 or control condition (scale bar = 100 μm). **h.** Fluorescent micrographs demonstrating adhesion between the HUVEC monolayer and the CT26 cells (green) treated with PP2 or control condition (scale bar = 100 μm). **i.** Western blot analysis of Src and p-Src expression in HCT116 cells treated with PYR-41 and Src inhibitor PP2. GAPDH is a loading control. **j.** Fluorescent micrographs showing adhesion between the HUVEC monolayer and the HCT116 cells (green) treated with PYR-41 and PP2 (scale bar = 100 μm). **k**. Quantification of the adhesion between the HUVEC monolayer and the HCT116 cells (N = 6, mean ± SD, Student's t-test, P = 0.004). **l.** F-actin labeling of membrane protrusions in HCT116 cells treated with PYR-41 and PP2 (scale bar = 100 μm). Arrows indicate extensions of cell membrane protrusions. **m.** Immunofluorescence staining of Talin and Vinculin in HCT116 cells treated with PYR-41 and PP2 (scale bar = 50 μm). Arrows indicate focal adhesions. **n.** Western blot analysis of p-FAK expression in HCT116 cells treated with propofol or control condition. GAPDH is a loading control. Ctrl, control; p-Src, phosphorylated Src; HUVEC, human umbilical vein endothelial cell; SD, standard deviation.

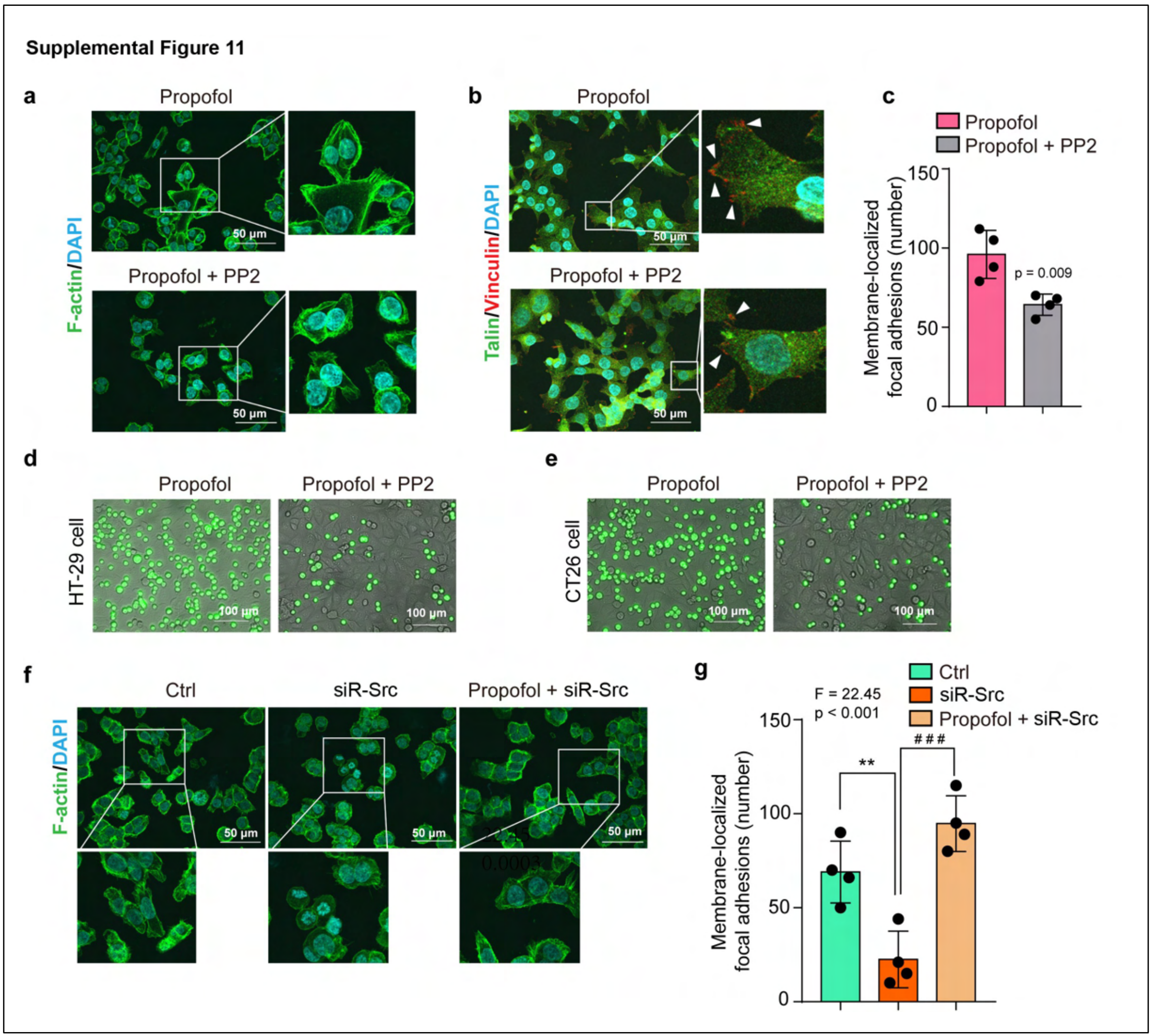


**Supplemental Figure 11. Src mediates the propofol-promoted adhesion and extension in HCT116 cells.**

**a.** F-actin labeling of membrane protrusions in propofol-treated HCT116 cells with or without PP2 treatment (scale bar = 50 μm). **b.** Immunofluorescence staining of Talin and Vinculin in propofol-treated HCT116 cells with or without PP2 treatment (scale bar = 50 μm). Arrows indicate focal adhesions. **c.** Quantification of the membrane-localized Talin and Vinculin in focal adhesions (N = 4, mean ± SD, Student's t-test, P = 0.009). **d.** Fluorescent micrographs demonstrating adhesion between the HUVEC monolayer and the propofol-treated HT-29 cells (green) with or without PP2 treatment (scale bar = 100 μm). **e.** Fluorescent micrographs demonstrating adhesion between the HUVEC monolayer and the propofol-treated CT26 cells (green) with or without PP2 treatment (scale bar = 100 μm). **f.** F-actin labeling of membrane protrusions in HCT116 cells with knockdown of Src and treated with propofol or control condition (scale bar = 50 μm). **g.** Quantification of the membrane-localized Talin and Vinculin in focal adhesions of HCT116 cells (N = 4, mean ± SD, One-way ANOVA test with post-hoc Bonferroni test, F = 22.45, P < 0.001). Ctrl, control; p-Src, phosphorylated Src; HUVEC, human umbilical vein endothelial cell; SD, standard deviation. ** P < 0.01, ### P < 0.001.

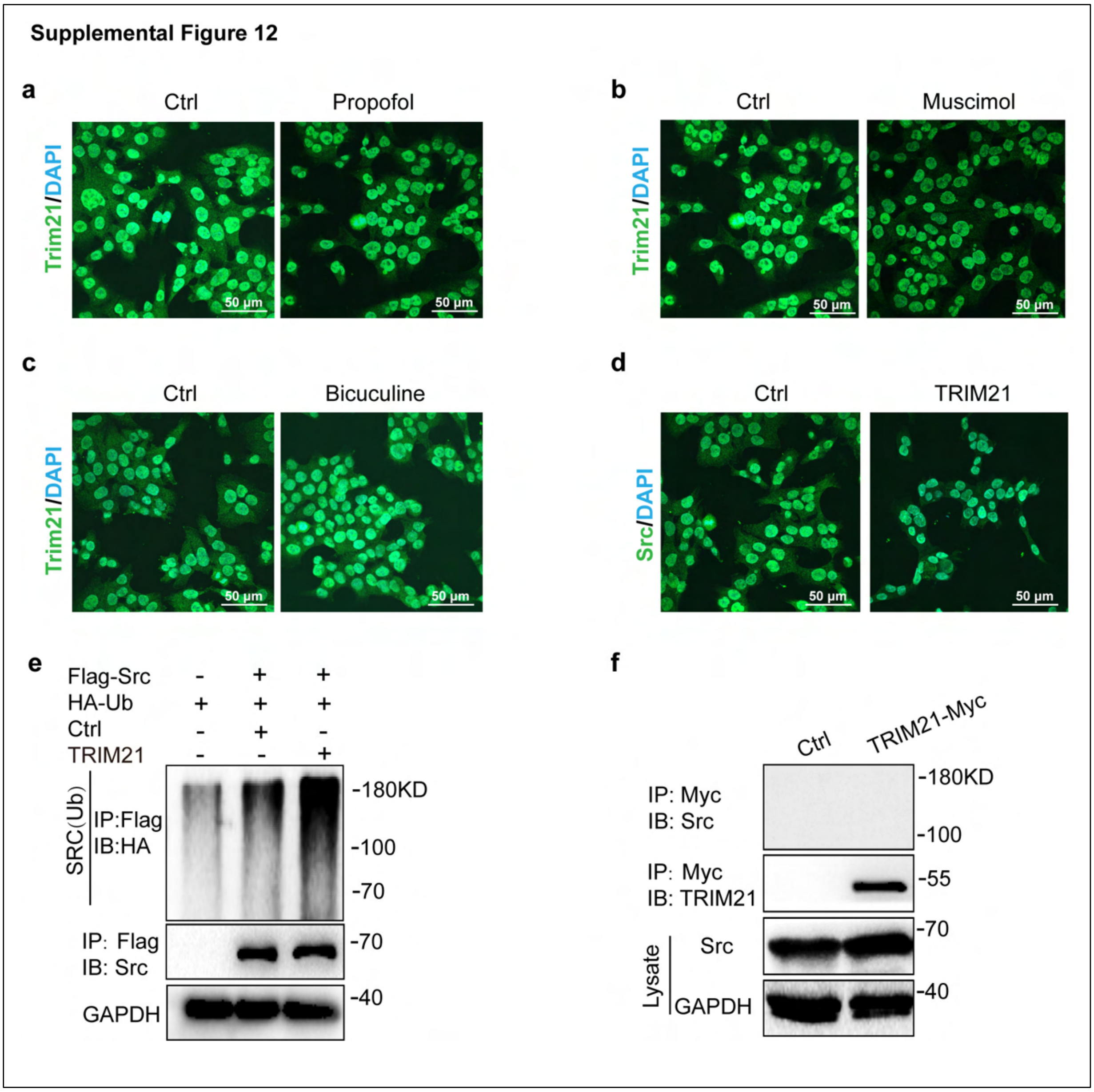


**Supplemental Figure 12. Propofol inhibits ubiquitylation of Src by TRIM21 through GABA$_A$R.**

Immunofluorescence staining of TRIM21 in HCT116 cells treated with (**a**) propofol, (**b**) muscimol, or (**c**) bicuculine (scale bar = 50 μm). **d.** Immunofluorescence staining of Src in HCT116 cells with overexpression of TRIM21 (scale bar = 50 μm). Ctrl, control. **e.** Western blot analysis of Src ubiquitylation in HCT116 cells overexpressing Src-Flag, TRIM21, or control vector. Src was immunoprecipitated with anti-Flag and immunoblotted with anti-HA. **f.** Co-IP assay did not demonstrate the direct interaction between E3 ligase-TRIM21 and Src. GAPDH is a loading control. Ctrl, control.

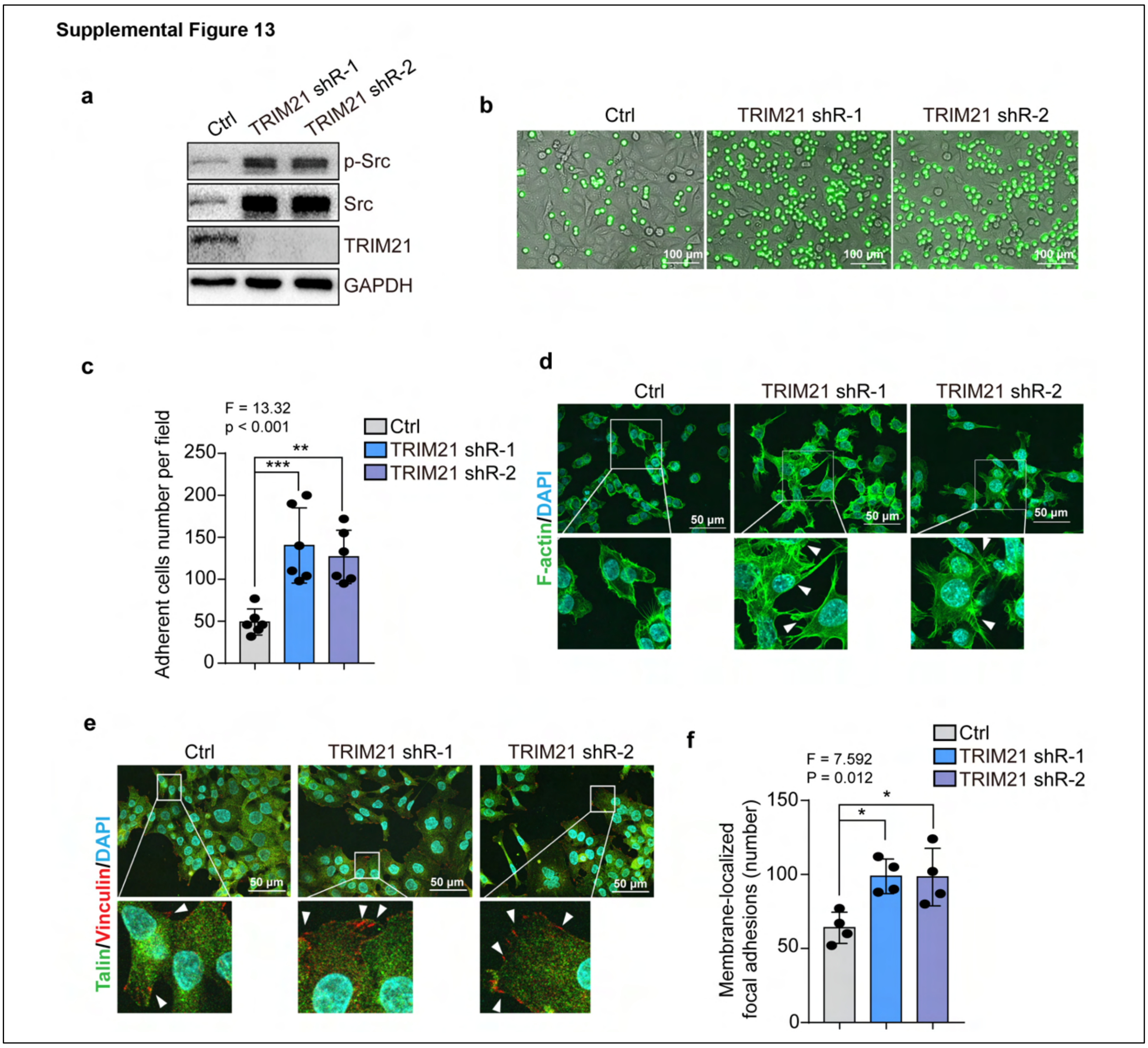


**Supplemental Figure 13. RNAi knockdown of TRIM21 enhances adhesion and extension in HCT116 cells.**

**a.** Western blot analysis of Src and p-Src expression following RNAi knockdown of TRIM21. GAPDH is a loading control. **b.** Fluorescent micrographs showing the adhesion between the HUVEC monolayer and the HCT116 cells with and without knockdown of TRIM21 (green) (scale bar = 100 μm). **c.** Quantification of the adhesion between HCT116 cells and HUVEC monolayer (N = 6, mean ± SD, One-way ANOVA test with post-hoc Bonferroni test, F = 13.32, P < 0.001). **d.** F-actin labeling of membrane protrusions in HCT116 cells following RNAi knockdown of TRIM21 (scale bar = 50 μm). Arrows indicate extensions of cell membrane protrusions. **e.** Immunofluorescence staining of Talin and Vinculin in HCT116 cells with or without knockdown of TRIM21 (scale bar = 50 μm). Arrows indicate focal adhesions. Ctrl, control. **f.** Quantification of the membrane-localized Talin and Vinculin in focal adhesions (N = 4, mean ± SD, One-way ANOVA test with post-hoc Bonferroni test, F = 7.592, P = 0.012). Ctrl, control; p-Src, phosphorylated Src; HUVEC, human umbilical vein endothelial cell; SD, standard deviation. * P < 0.05, ** P < 0.01, *** P < 0.001.

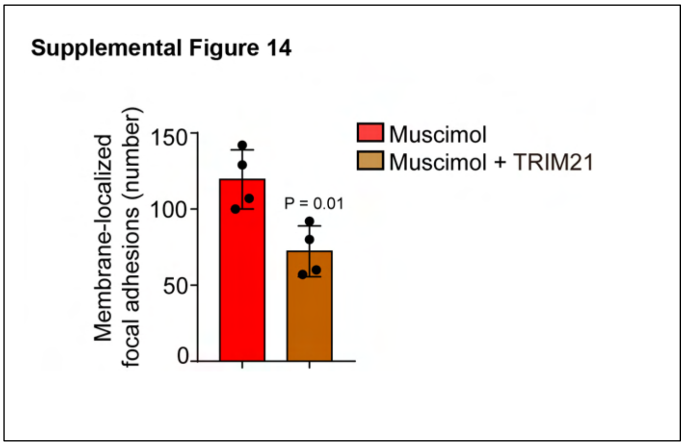


**Supplemental Figure 14. Overexpression of TRIM21 attenuates the muscimol-induced enhancement of HCT116 cells adhesion and extension.**

Quantification of the membrane-localized Talin and Vinculin in focal adhesions (N = 4, mean ± SD, Student's t-test, $P = 0.01$). SD, standard deviation.
